# Supplementary figures and images for: Purging viral latency by a bifunctional HSV-vectored therapeutic vaccine in chronically SIV-infected macaques
Source: eLife. 2025 Apr 23;13:RP95964. doi: 10.7554/eLife.95964 (PMC12017772; doi:10.7554/eLife.95964)

flag

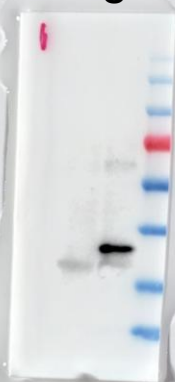

gapdh

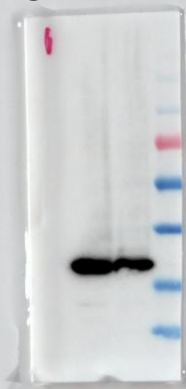

Supplement: Figure 1—source data 1. [file elife-95964-fig1-data1.pdf]

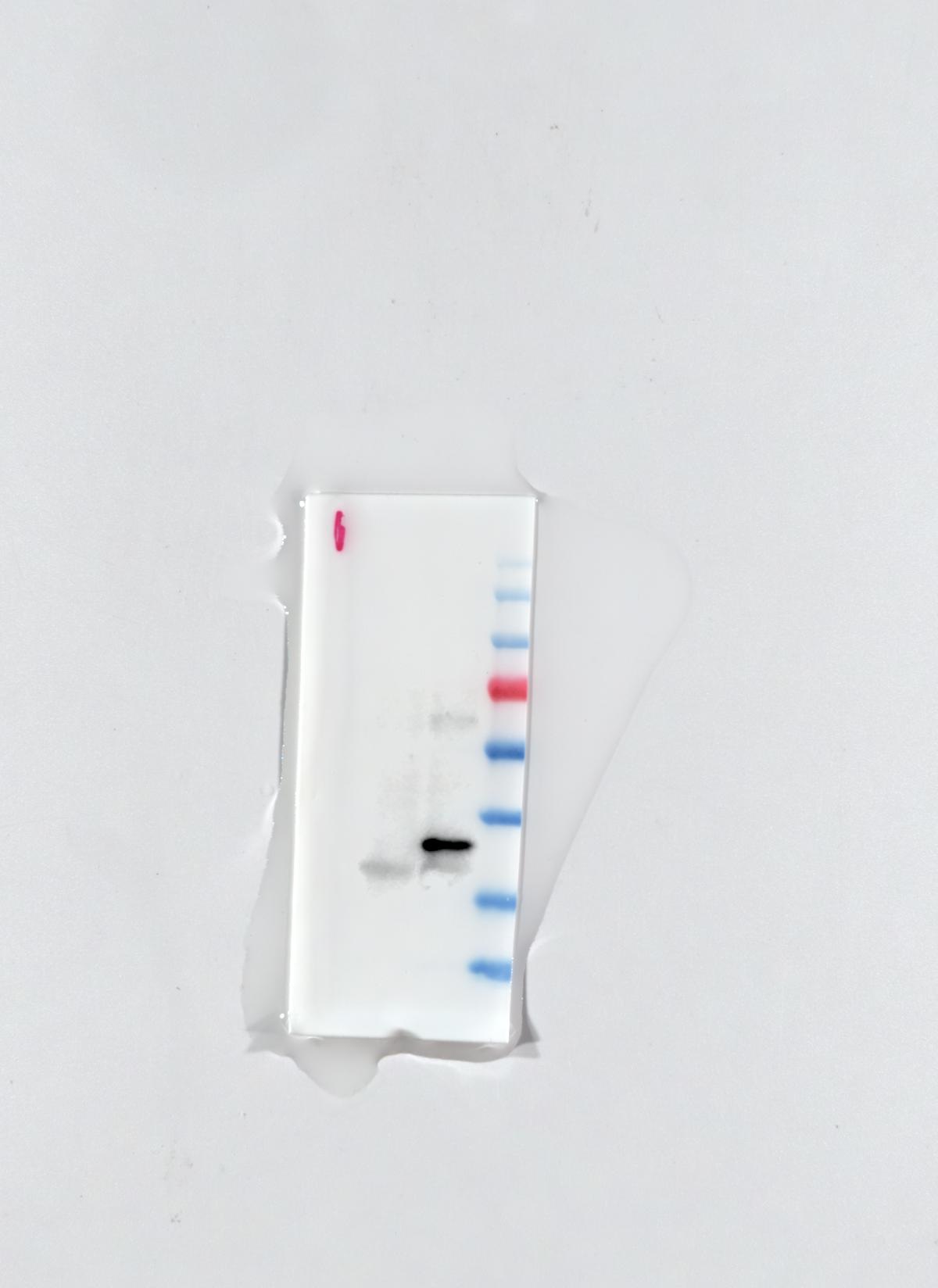

Supplement: Figure 1—source data 2. [file elife-95964-fig1-data2.zip › Figure 1-source data 2/flag.jpg]

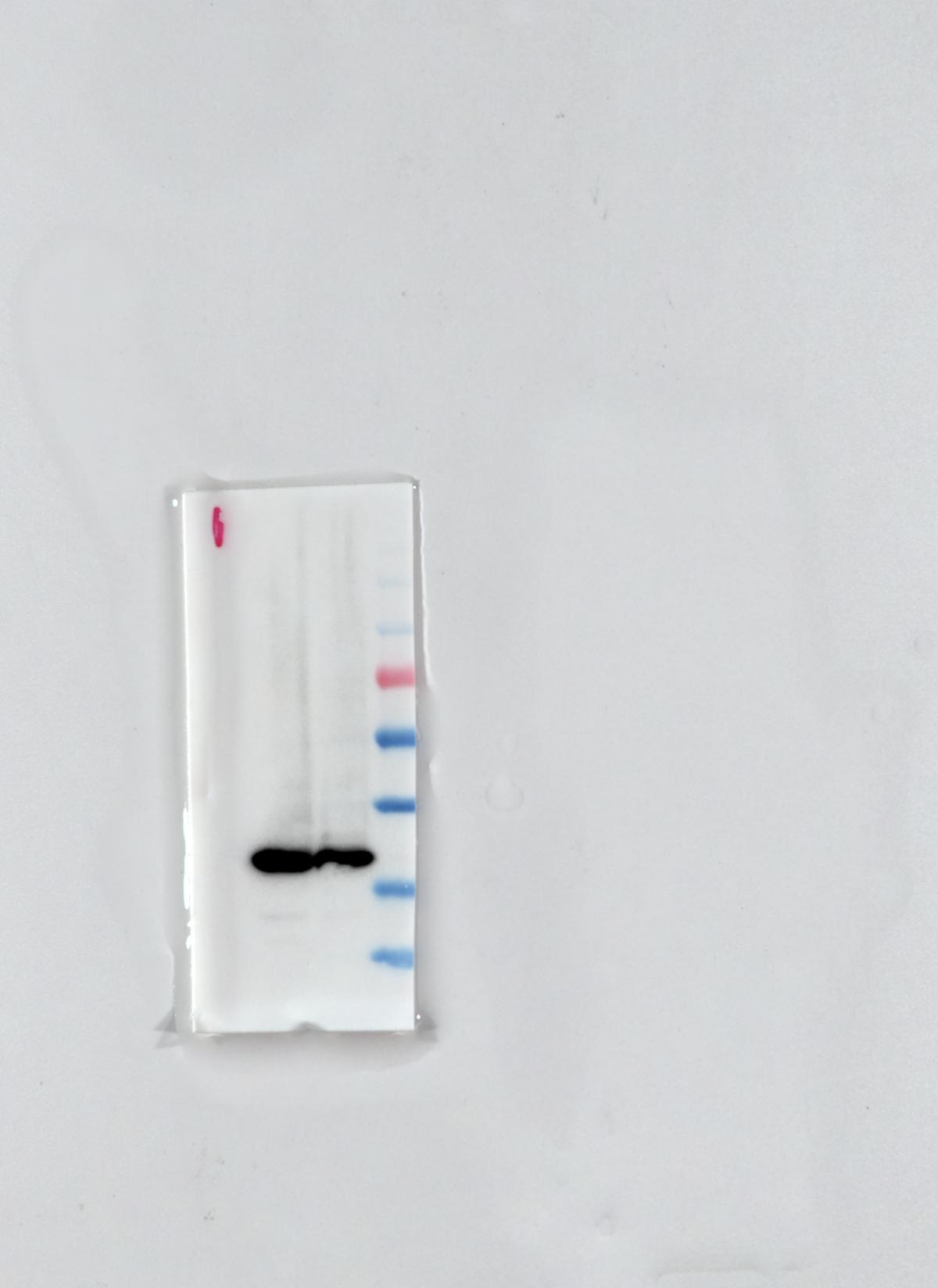

Supplement: Figure 1—source data 2. [file elife-95964-fig1-data2.zip › Figure 1-source data 2/GAPDH.jpg]

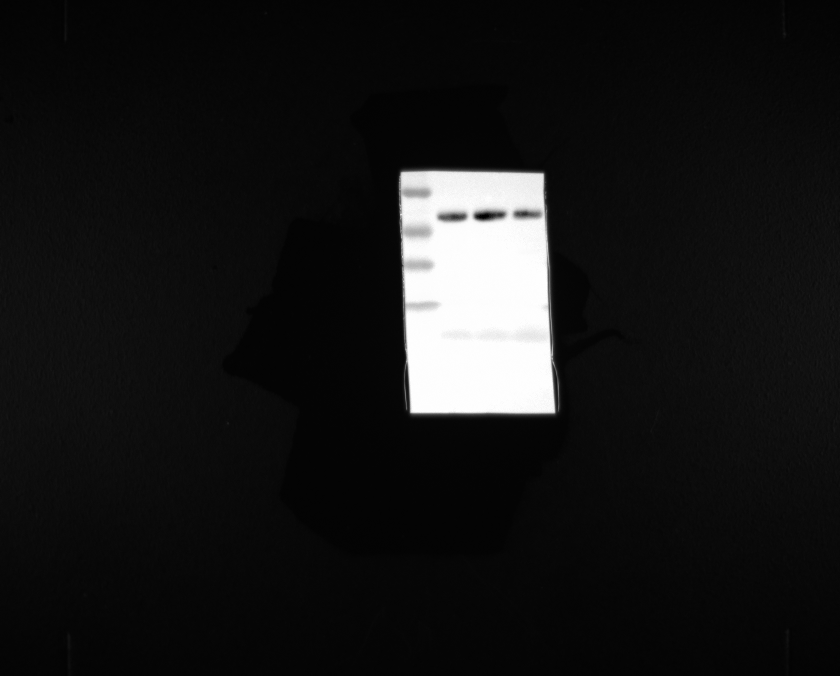

Supplement: Figure 2—source data 2. [file elife-95964-fig2-data2.zip › Figure 2-source data 2/figure 2a/gapdh.tif]

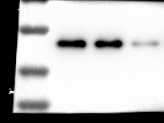

Supplement: Figure 2—source data 2. [file elife-95964-fig2-data2.zip › Figure 2-source data 2/figure 2a/IKBa.tif]

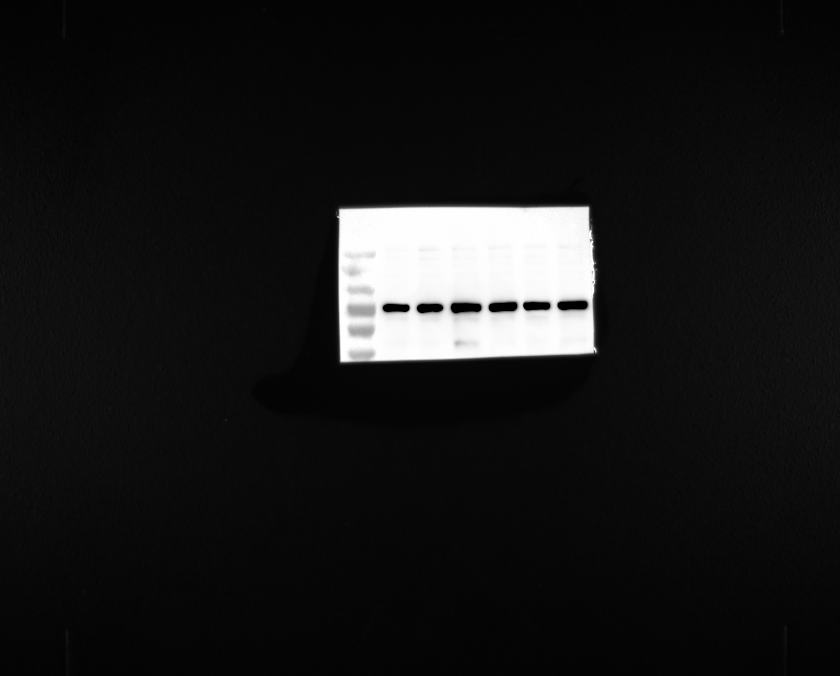

Supplement: Figure 2—source data 2. [file elife-95964-fig2-data2.zip › Figure 2-source data 2/figure 2a/laminb.tif]

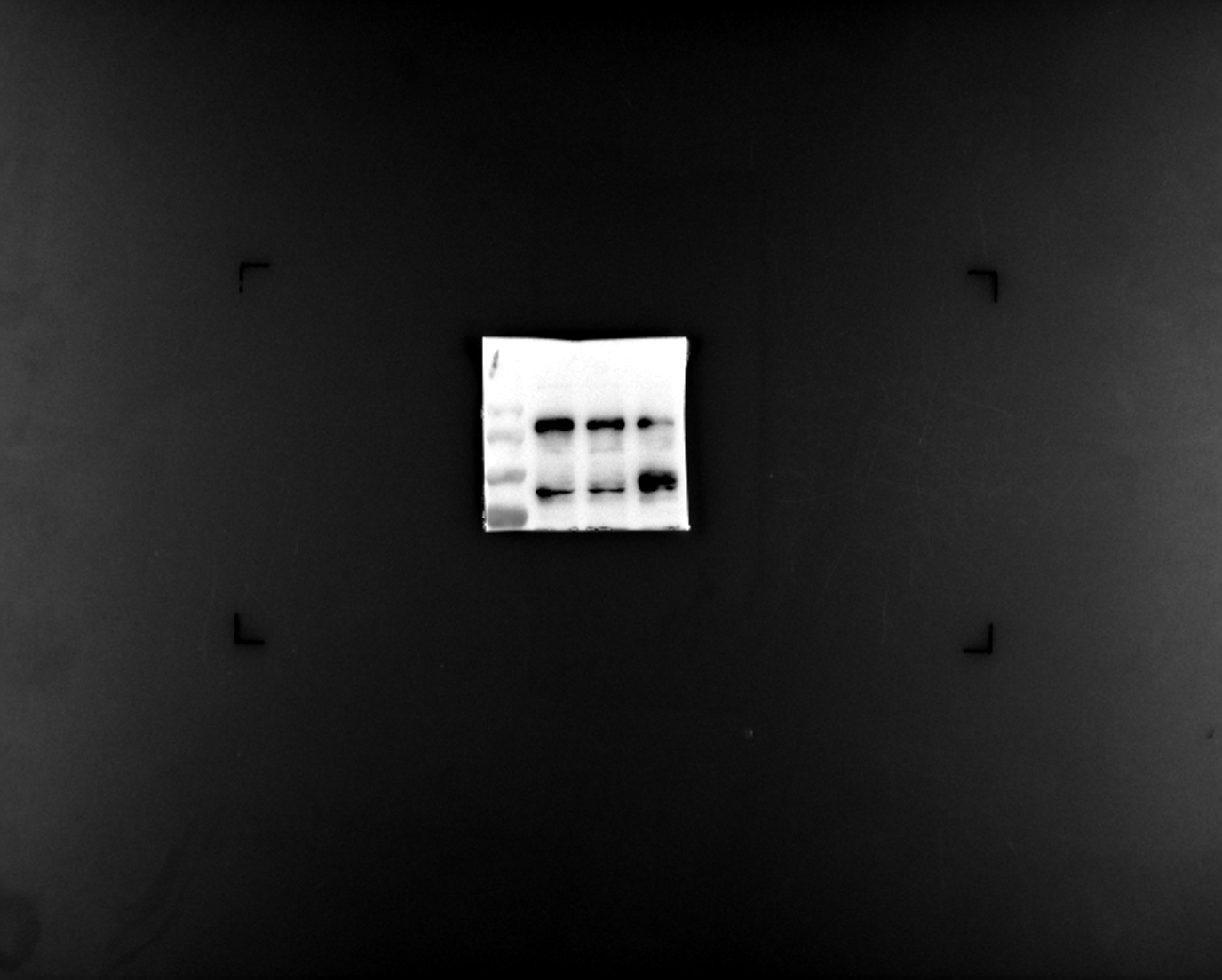

Supplement: Figure 2—source data 2. [file elife-95964-fig2-data2.zip › Figure 2-source data 2/figure 2a/p-ikk.jpg]

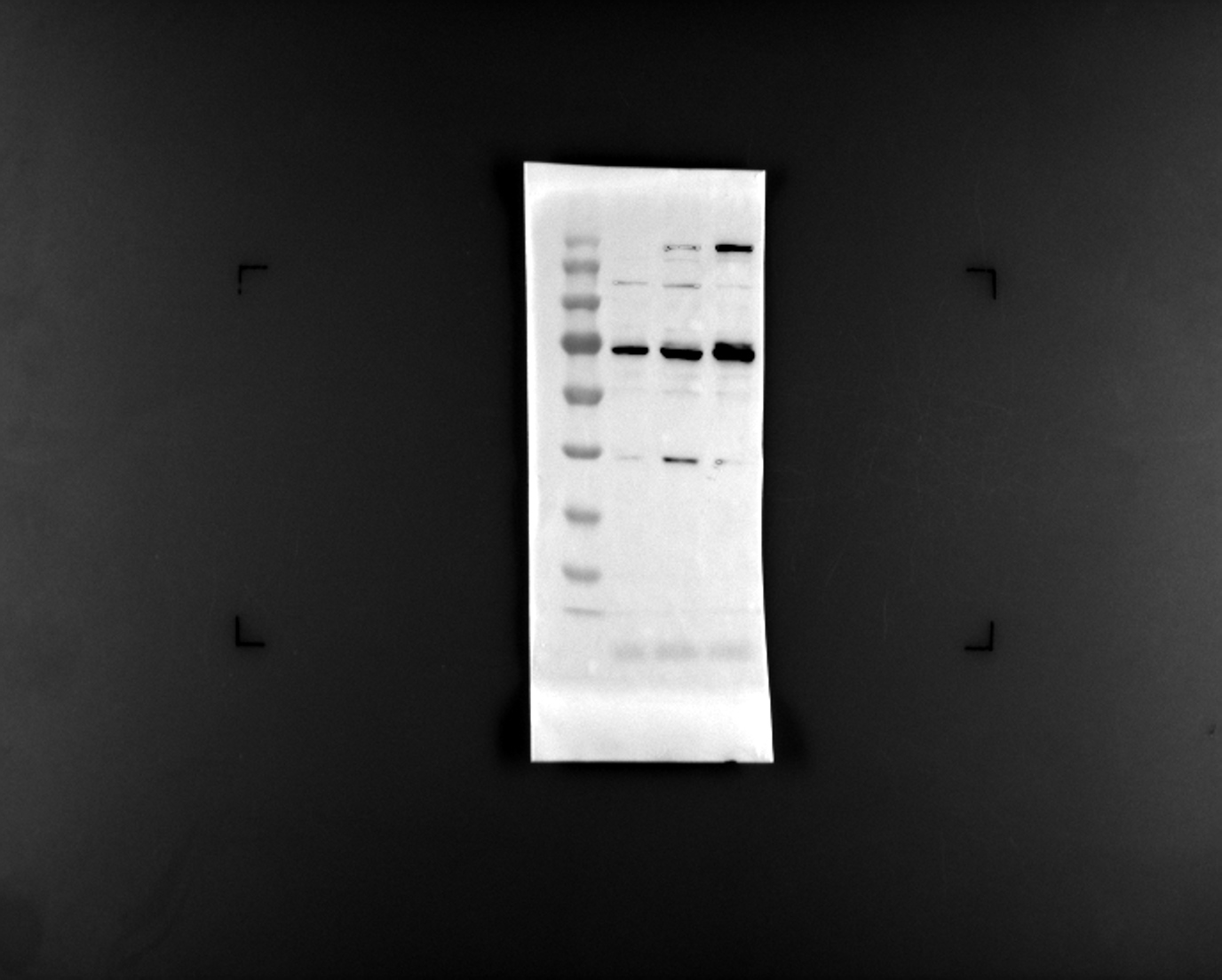

Supplement: Figure 2—source data 2. [file elife-95964-fig2-data2.zip › Figure 2-source data 2/figure 2a/p65.jpg]

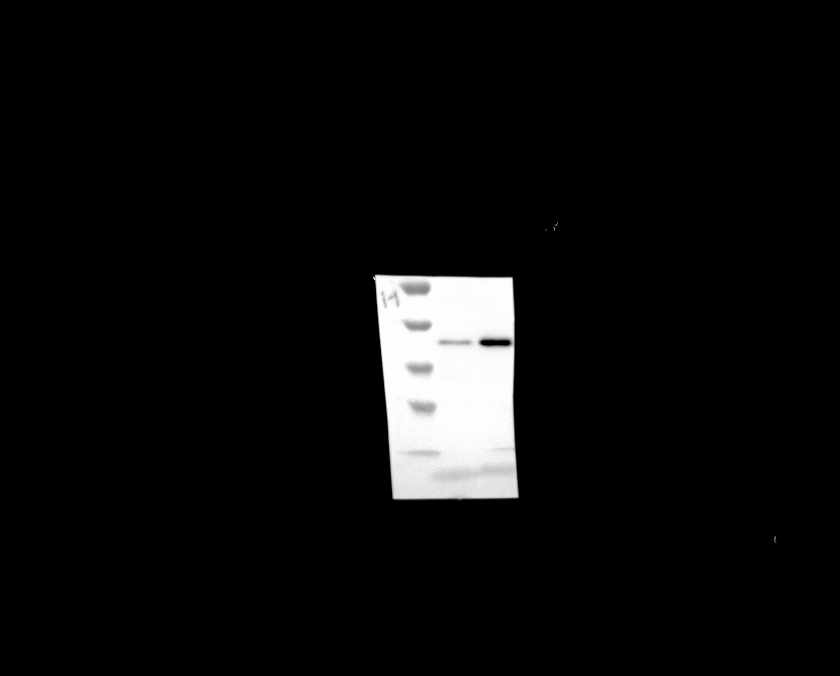

Supplement: Figure 2—source data 2. [file elife-95964-fig2-data2.zip › Figure 2-source data 2/figure 2b/FLAG-input-left.tif]

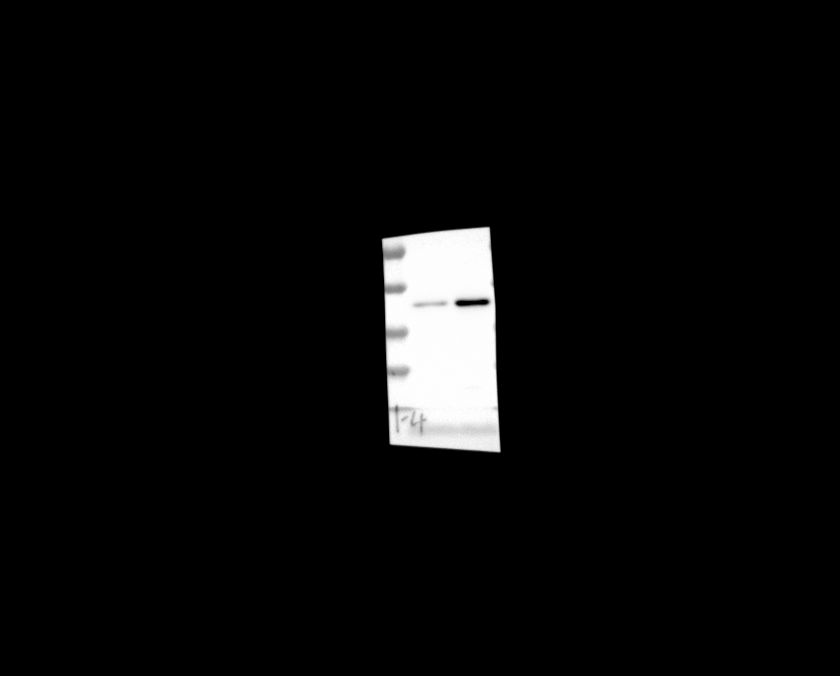

Supplement: Figure 2—source data 2. [file elife-95964-fig2-data2.zip › Figure 2-source data 2/figure 2b/Flag-input-right.tif]

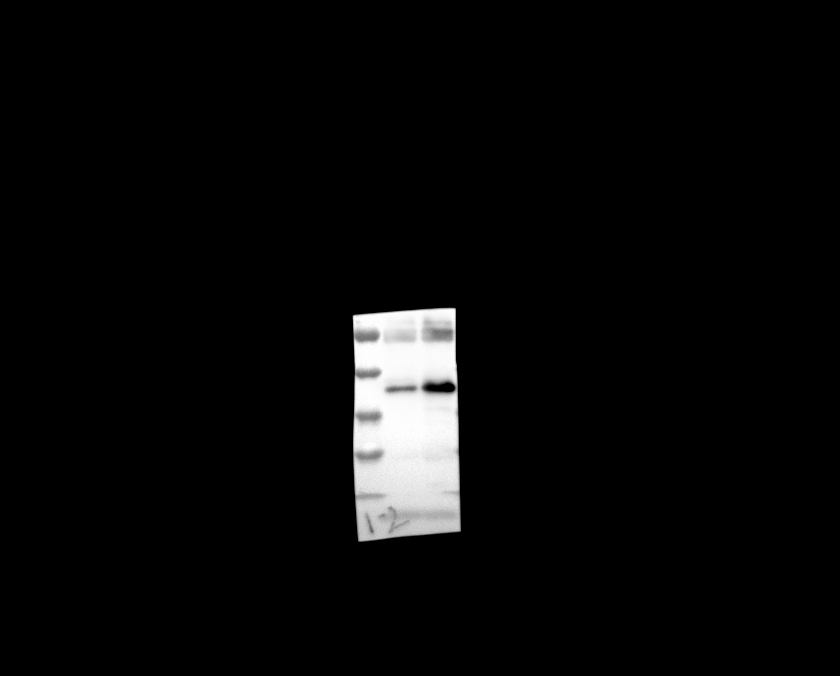

Supplement: Figure 2—source data 2. [file elife-95964-fig2-data2.zip › Figure 2-source data 2/figure 2b/FLAG-ip-left.tif]

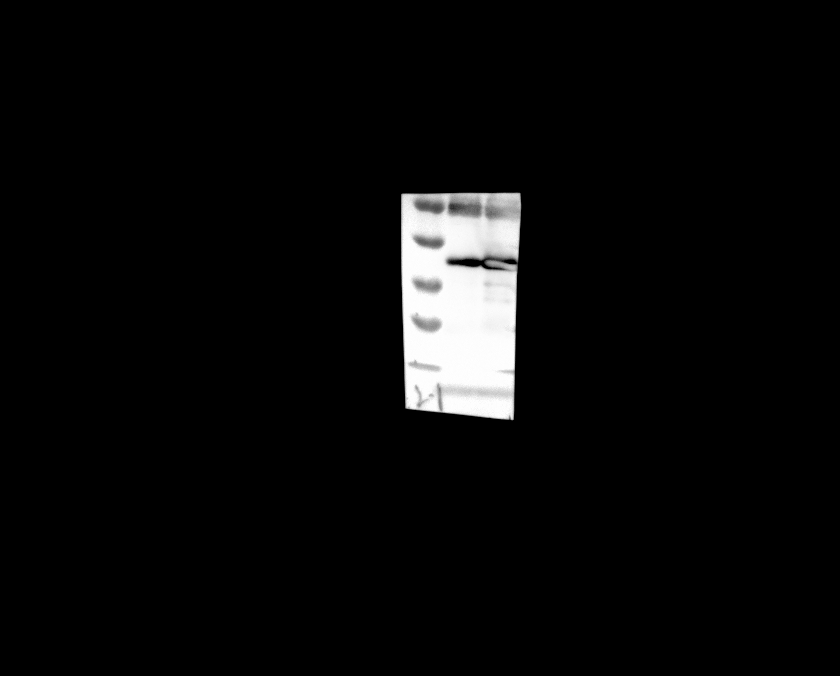

Supplement: Figure 2—source data 2. [file elife-95964-fig2-data2.zip › Figure 2-source data 2/figure 2b/flag-ip-right.tif]

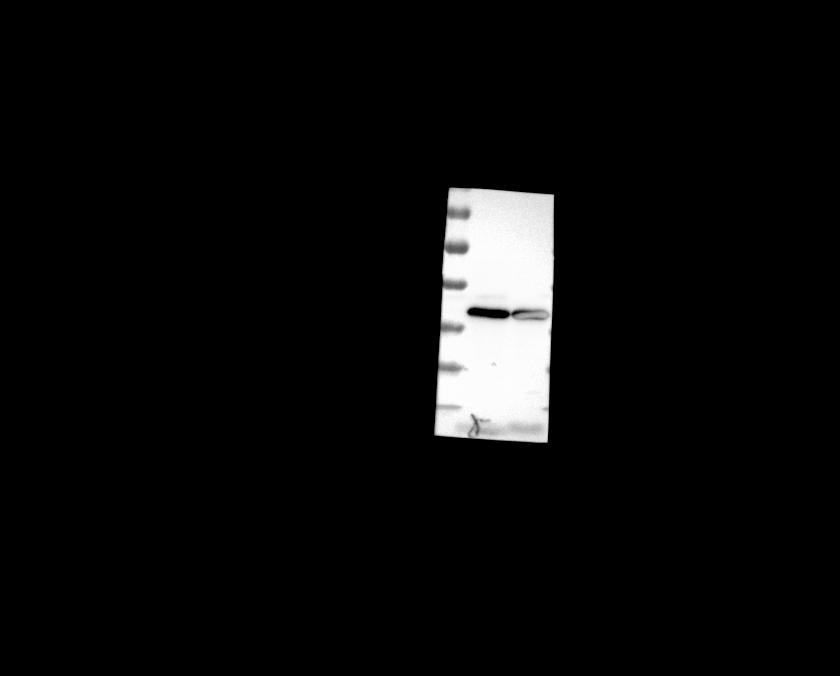

Supplement: Figure 2—source data 2. [file elife-95964-fig2-data2.zip › Figure 2-source data 2/figure 2b/GAPDH-left.tif]

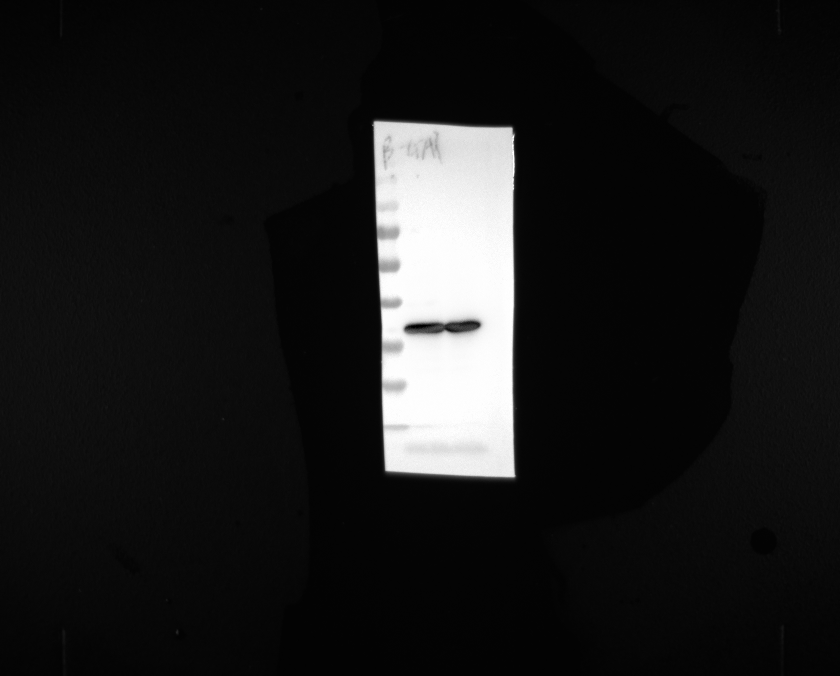

Supplement: Figure 2—source data 2. [file elife-95964-fig2-data2.zip › Figure 2-source data 2/figure 2b/GAPDH-right.tif]

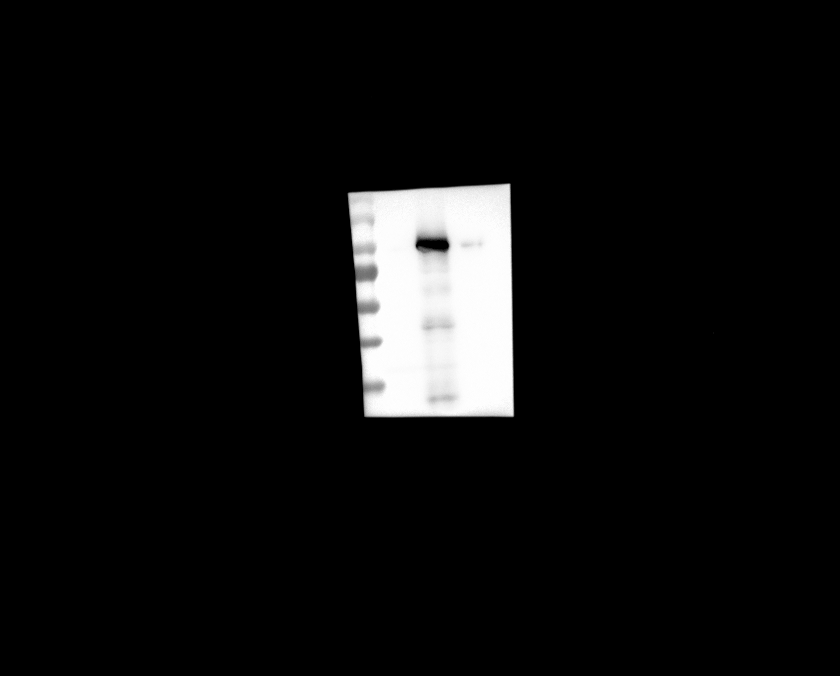

Supplement: Figure 2—source data 2. [file elife-95964-fig2-data2.zip › Figure 2-source data 2/figure 2b/HA-input-left.tif]

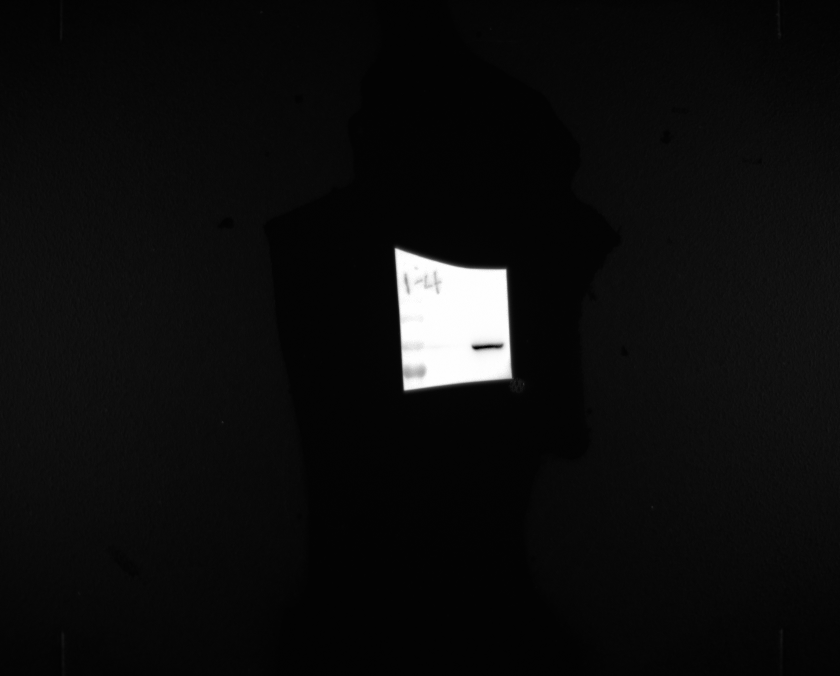

Supplement: Figure 2—source data 2. [file elife-95964-fig2-data2.zip › Figure 2-source data 2/figure 2b/HA-input-right.tif]

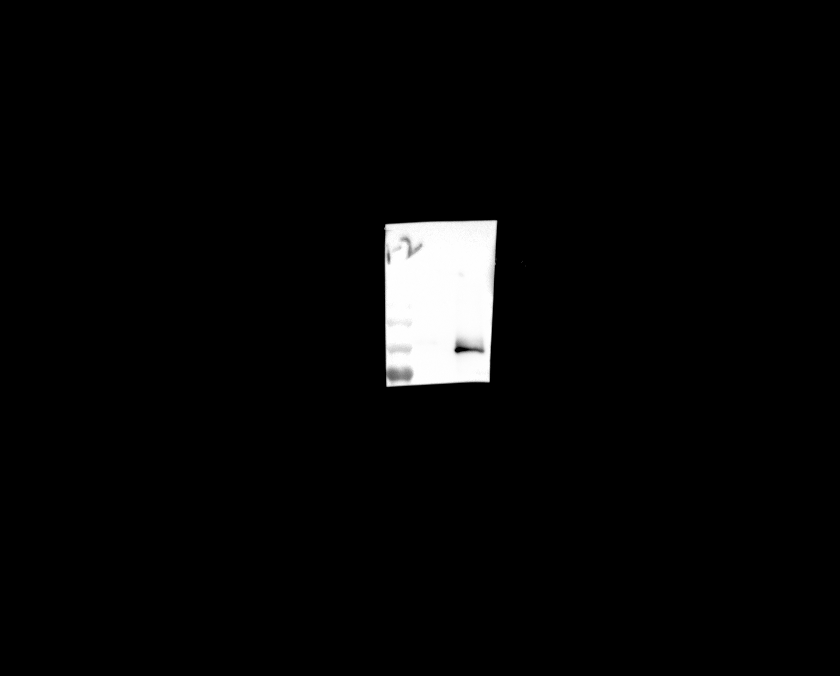

Supplement: Figure 2—source data 2. [file elife-95964-fig2-data2.zip › Figure 2-source data 2/figure 2b/HA-IP-left.tif]

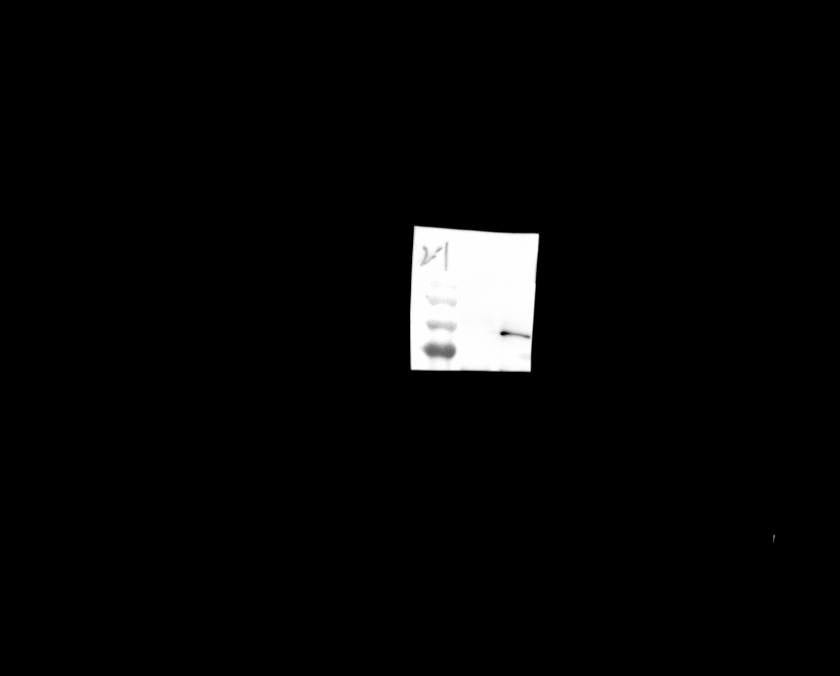

Supplement: Figure 2—source data 2. [file elife-95964-fig2-data2.zip › Figure 2-source data 2/figure 2b/HA-IP-right.tif]

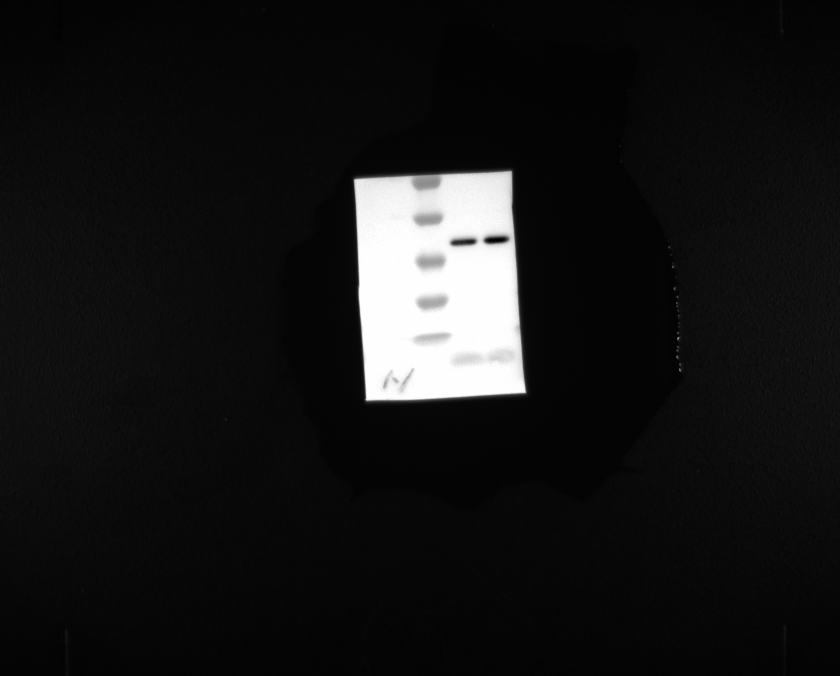

Supplement: Figure 2—source data 2. [file elife-95964-fig2-data2.zip › Figure 2-source data 2/figure 2c/GAPDH.tif]

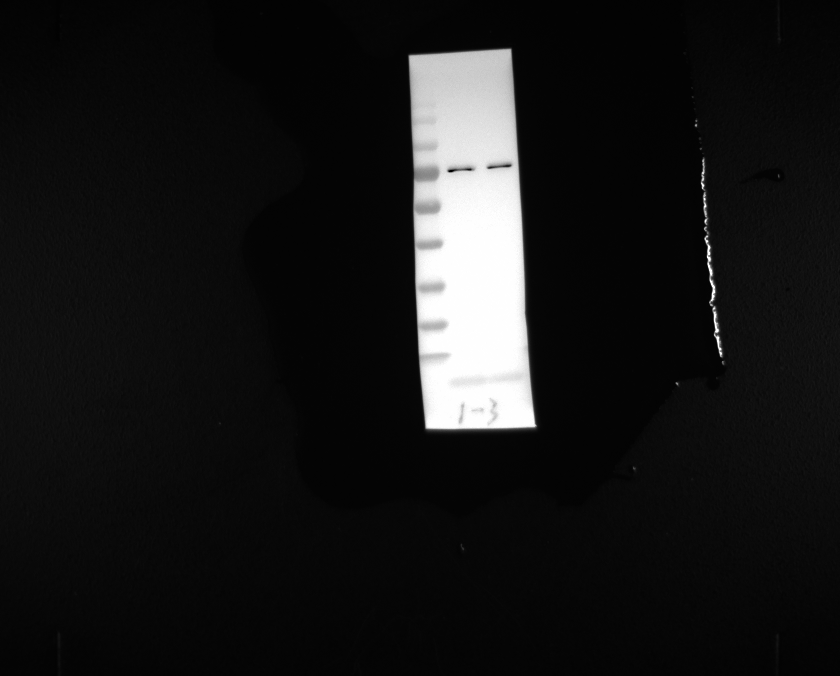

Supplement: Figure 2—source data 2. [file elife-95964-fig2-data2.zip › Figure 2-source data 2/figure 2c/lamin B.tif]

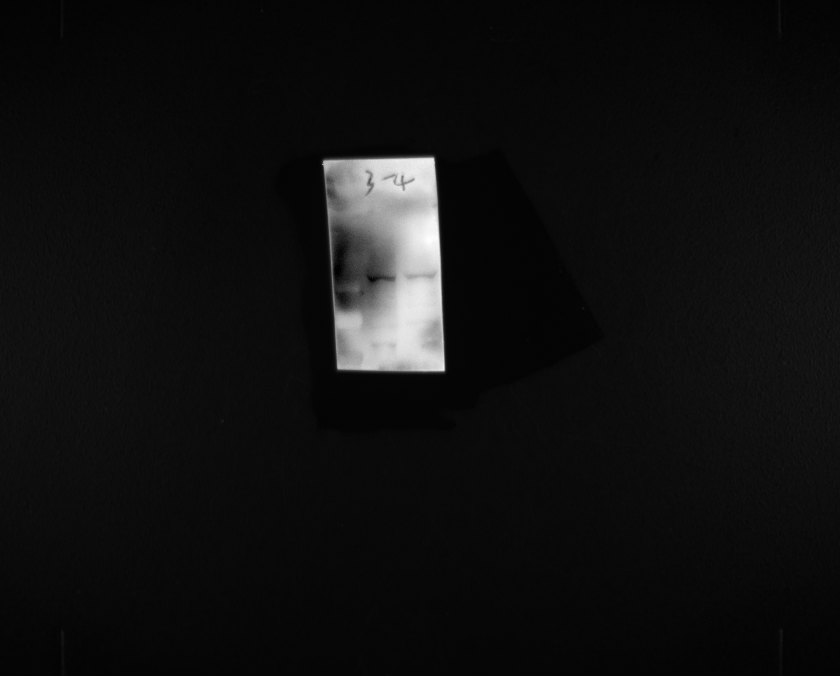

Supplement: Figure 2—source data 2. [file elife-95964-fig2-data2.zip › Figure 2-source data 2/figure 2c/NF-kb p65.tif]

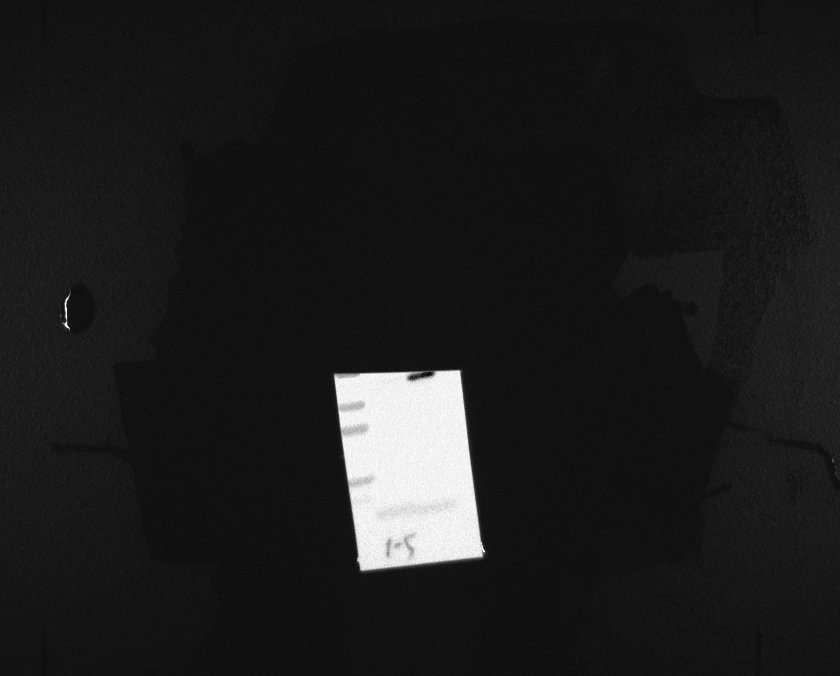

Supplement: Figure 2—source data 2. [file elife-95964-fig2-data2.zip › Figure 2-source data 2/figure 2g/left/flag-input.tif]

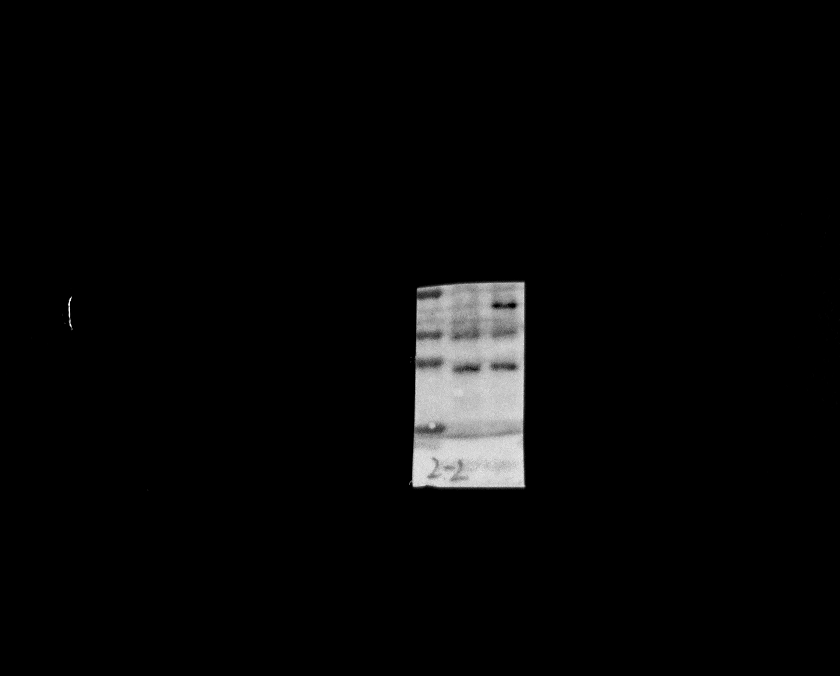

Supplement: Figure 2—source data 2. [file elife-95964-fig2-data2.zip › Figure 2-source data 2/figure 2g/left/flag-ip.tif]

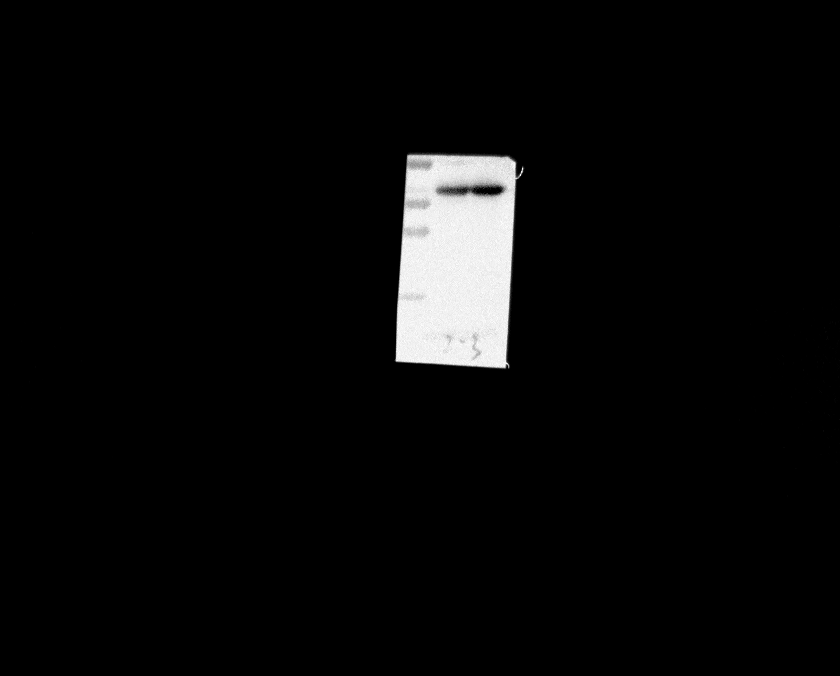

Supplement: Figure 2—source data 2. [file elife-95964-fig2-data2.zip › Figure 2-source data 2/figure 2g/left/gapdh.tif]

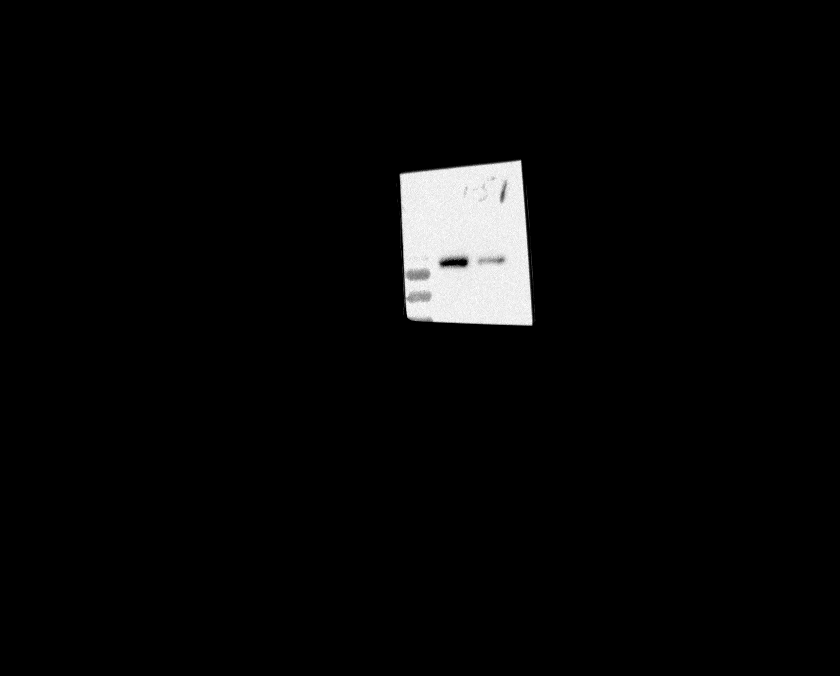

Supplement: Figure 2—source data 2. [file elife-95964-fig2-data2.zip › Figure 2-source data 2/figure 2g/left/myc-input.tif]

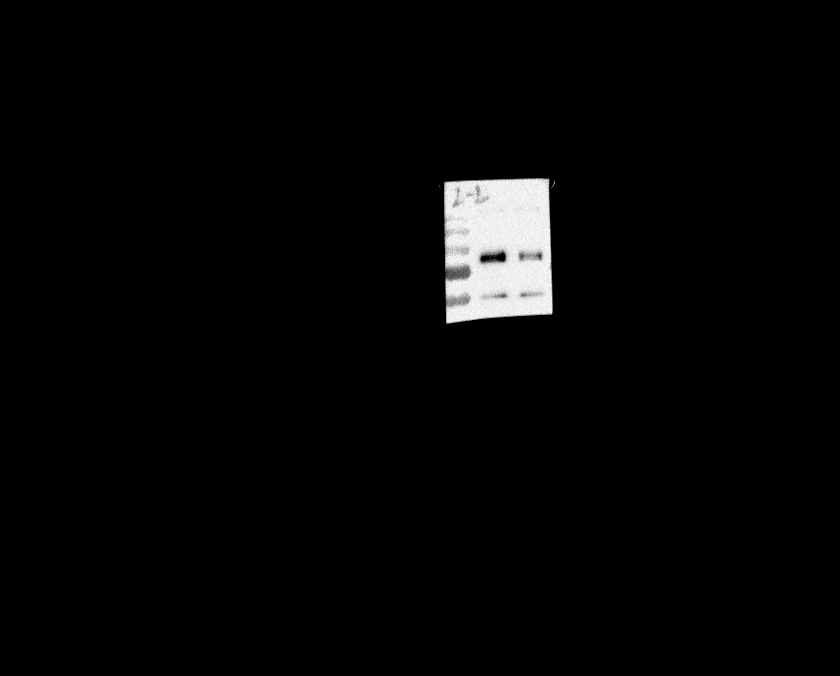

Supplement: Figure 2—source data 2. [file elife-95964-fig2-data2.zip › Figure 2-source data 2/figure 2g/left/myc-ip.tif]

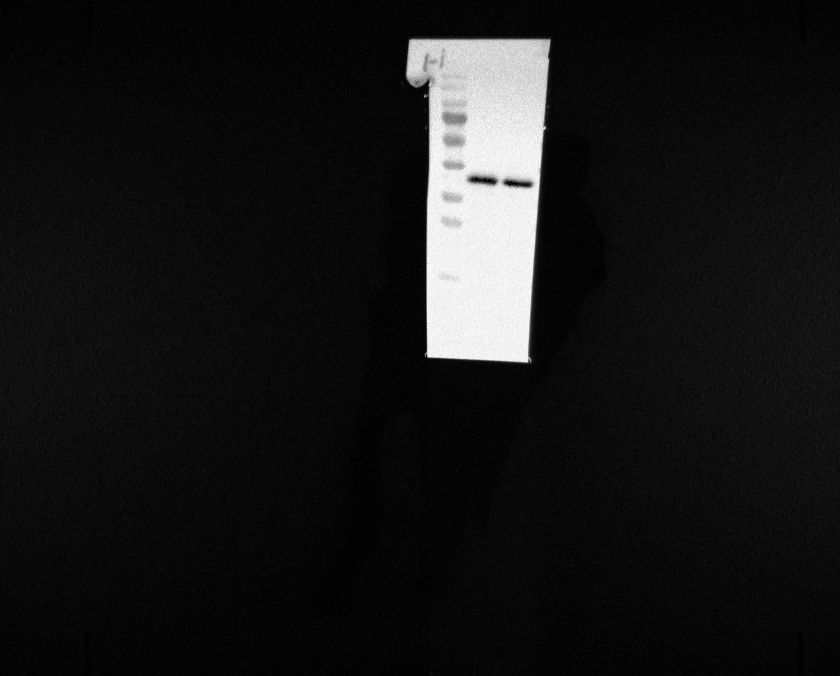

Supplement: Figure 2—source data 2. [file elife-95964-fig2-data2.zip › Figure 2-source data 2/figure 2g/right/flag-input.tif]

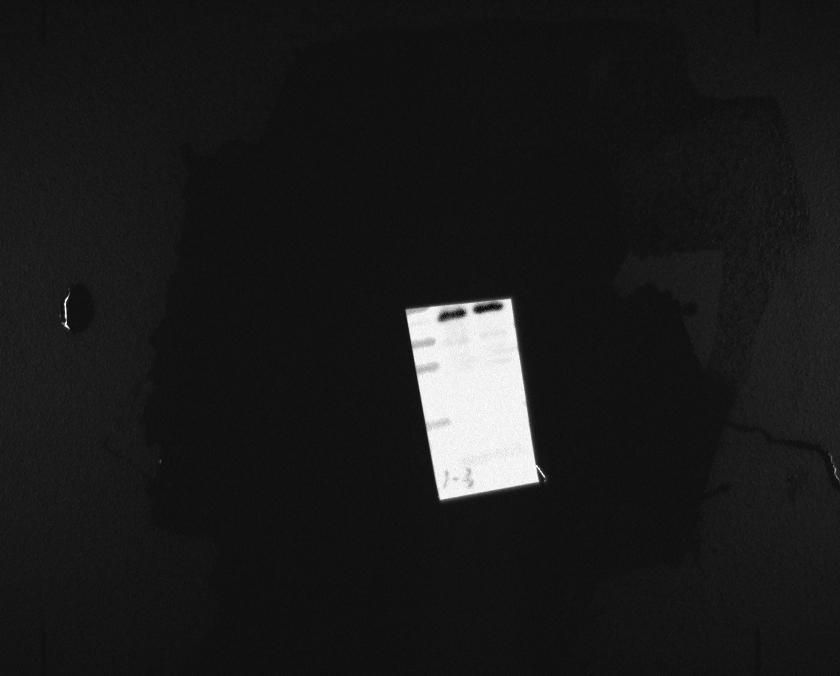

Supplement: Figure 2—source data 2. [file elife-95964-fig2-data2.zip › Figure 2-source data 2/figure 2g/right/flag-ip.tif]

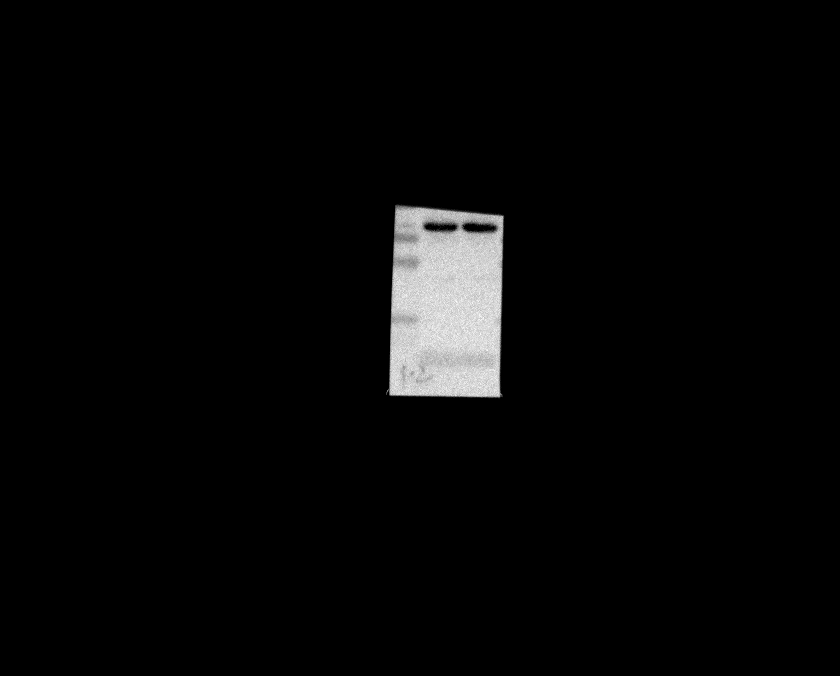

Supplement: Figure 2—source data 2. [file elife-95964-fig2-data2.zip › Figure 2-source data 2/figure 2g/right/gapdh.tif]

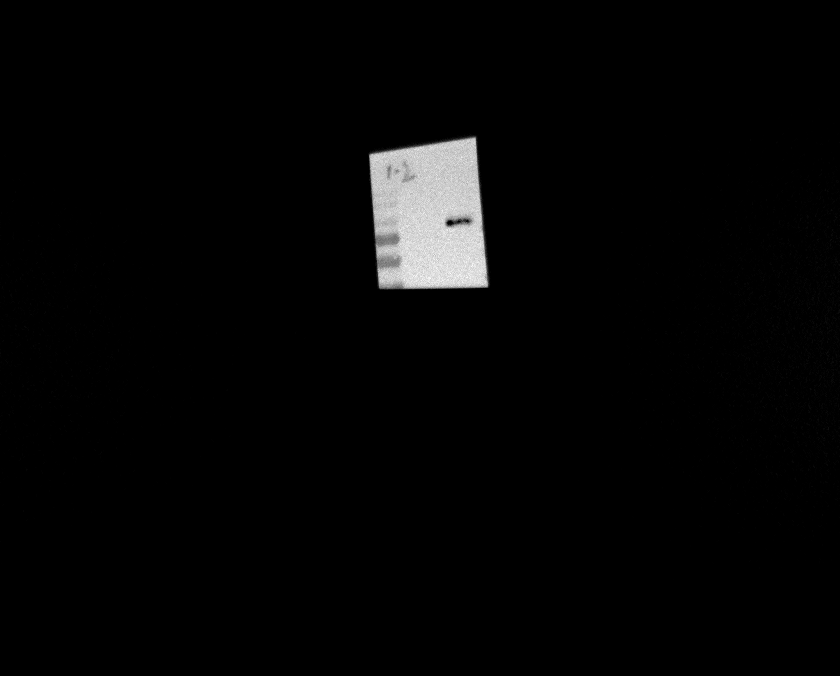

Supplement: Figure 2—source data 2. [file elife-95964-fig2-data2.zip › Figure 2-source data 2/figure 2g/right/myc-input.tif]

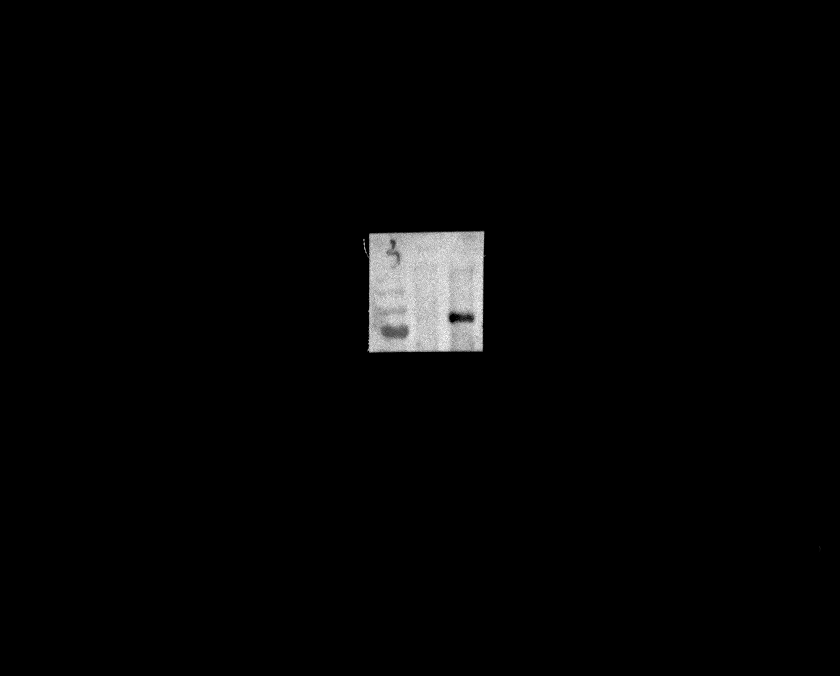

Supplement: Figure 2—source data 2. [file elife-95964-fig2-data2.zip › Figure 2-source data 2/figure 2g/right/myc-ip.tif]

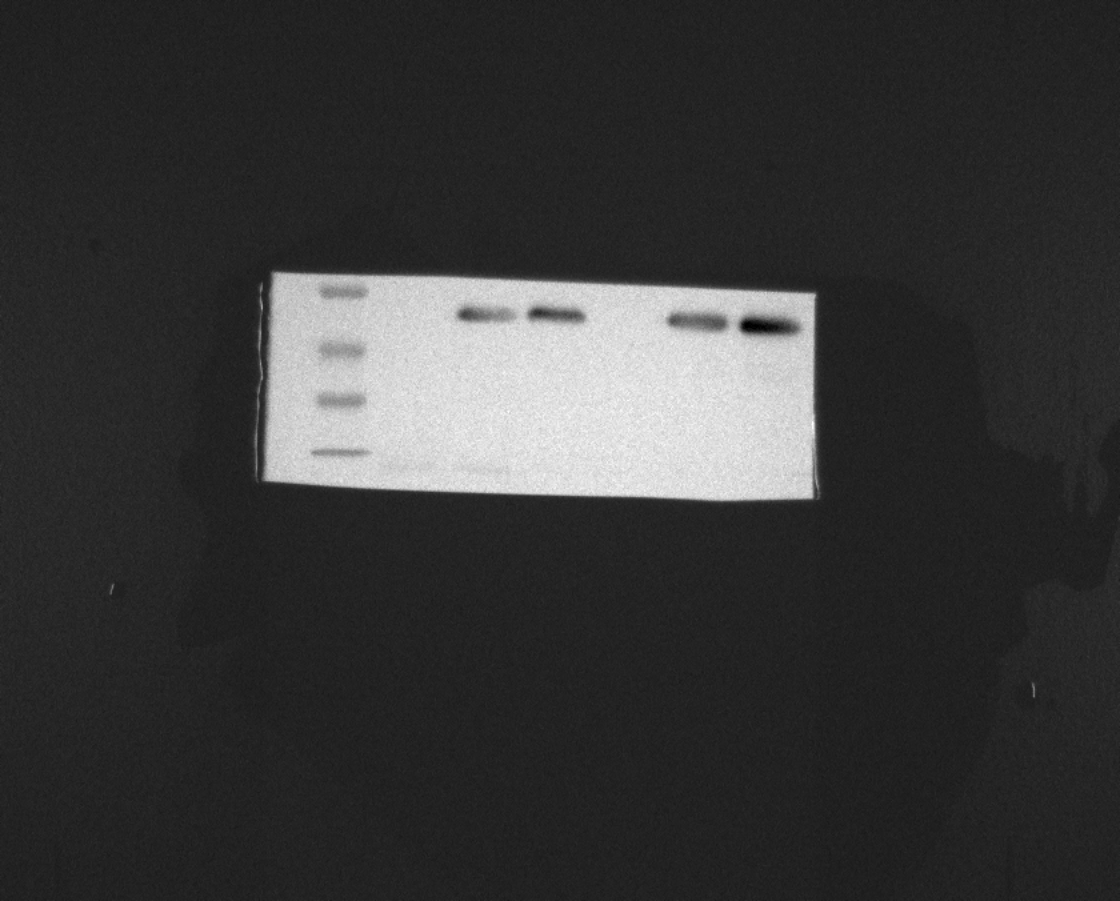

Supplement: Figure 2—source data 2. [file elife-95964-fig2-data2.zip › Figure 2-source data 2/figure 2h/Flag.tif]

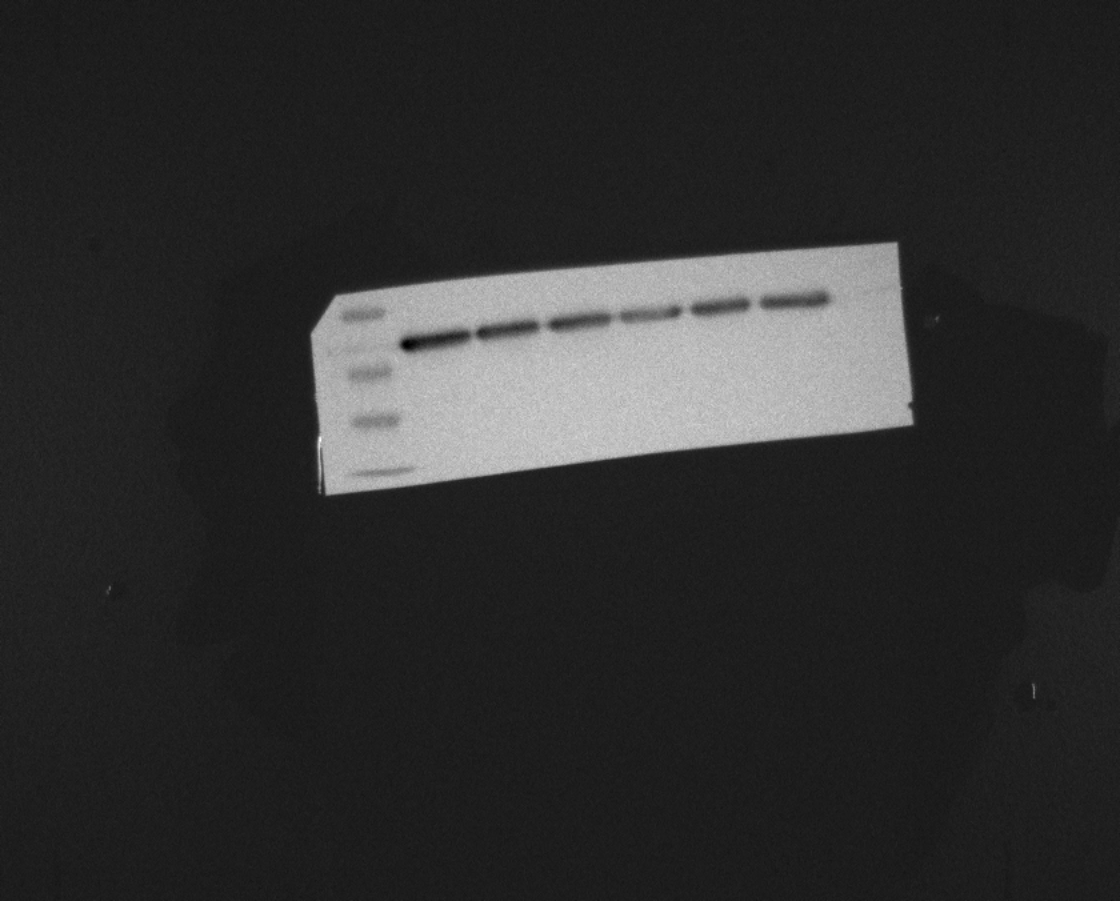

Supplement: Figure 2—source data 2. [file elife-95964-fig2-data2.zip › Figure 2-source data 2/figure 2h/GAPDH.tif]

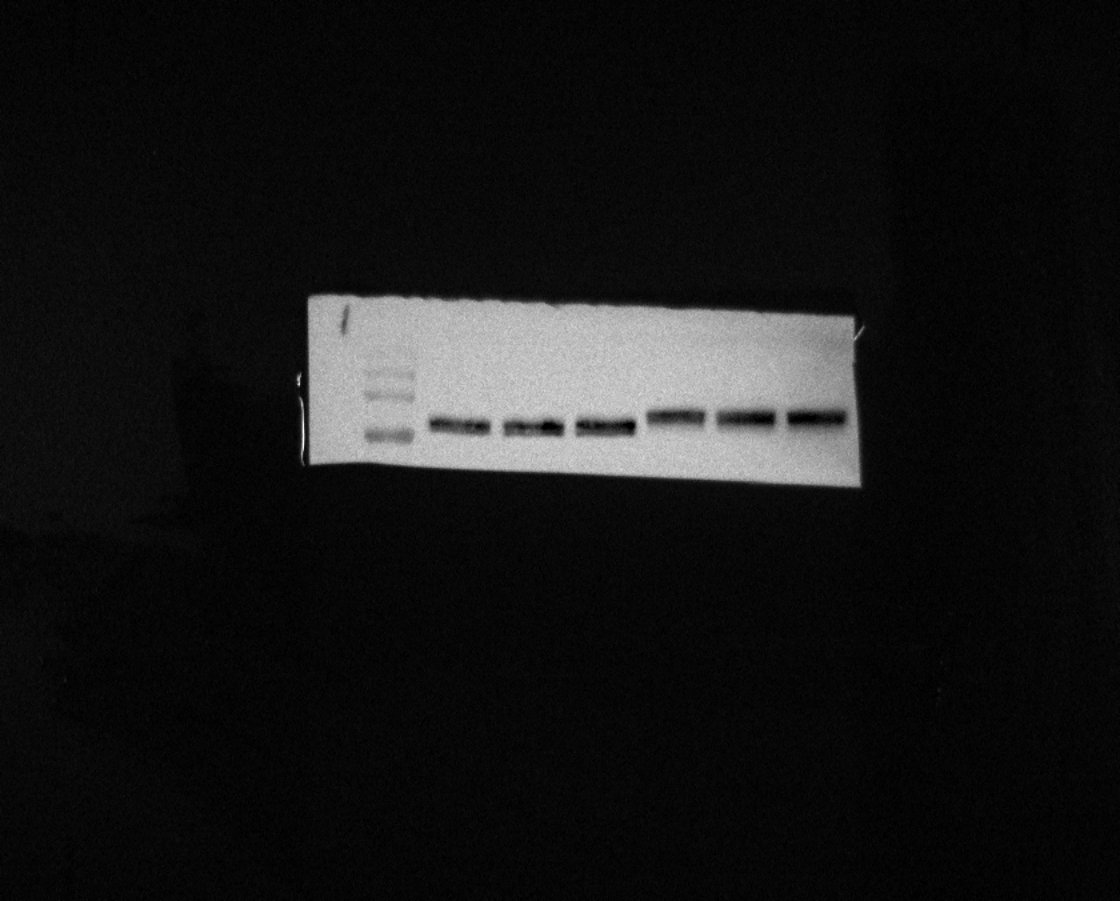

Supplement: Figure 2—source data 2. [file elife-95964-fig2-data2.zip › Figure 2-source data 2/figure 2h/HSF1.tif]

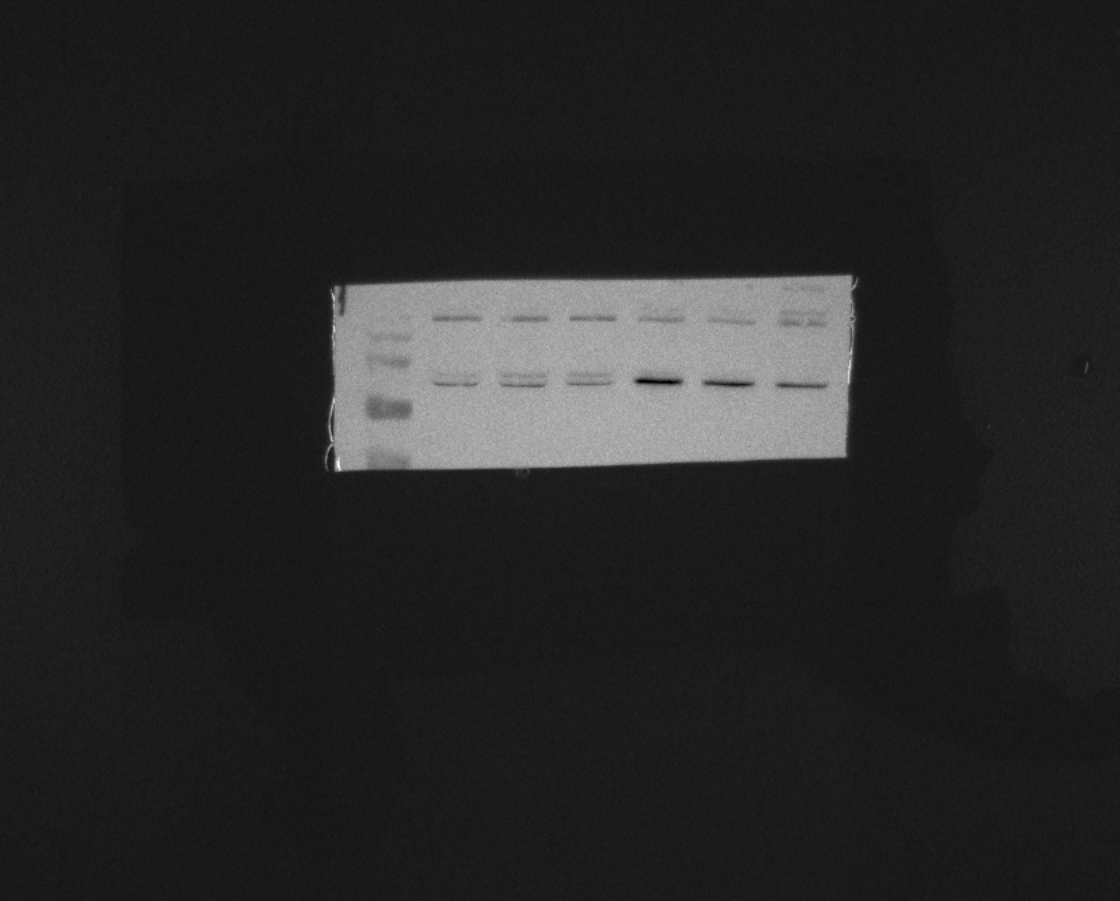

Supplement: Figure 2—source data 2. [file elife-95964-fig2-data2.zip › Figure 2-source data 2/figure 2h/P-HSF1 (Ser 320).tif]

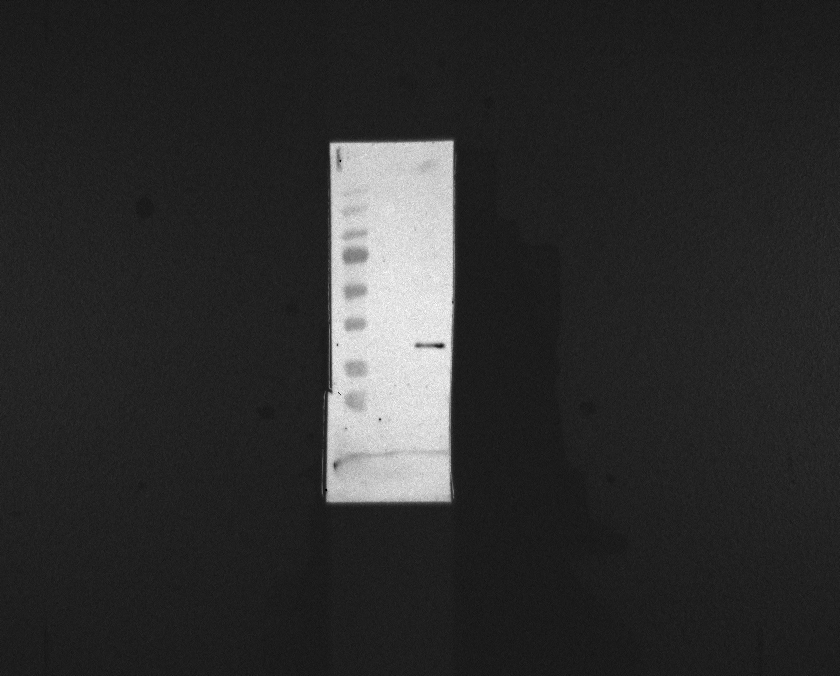

Supplement: Figure 2—source data 2. [file elife-95964-fig2-data2.zip › Figure 2-source data 2/figure 2i/flag-input.tif]

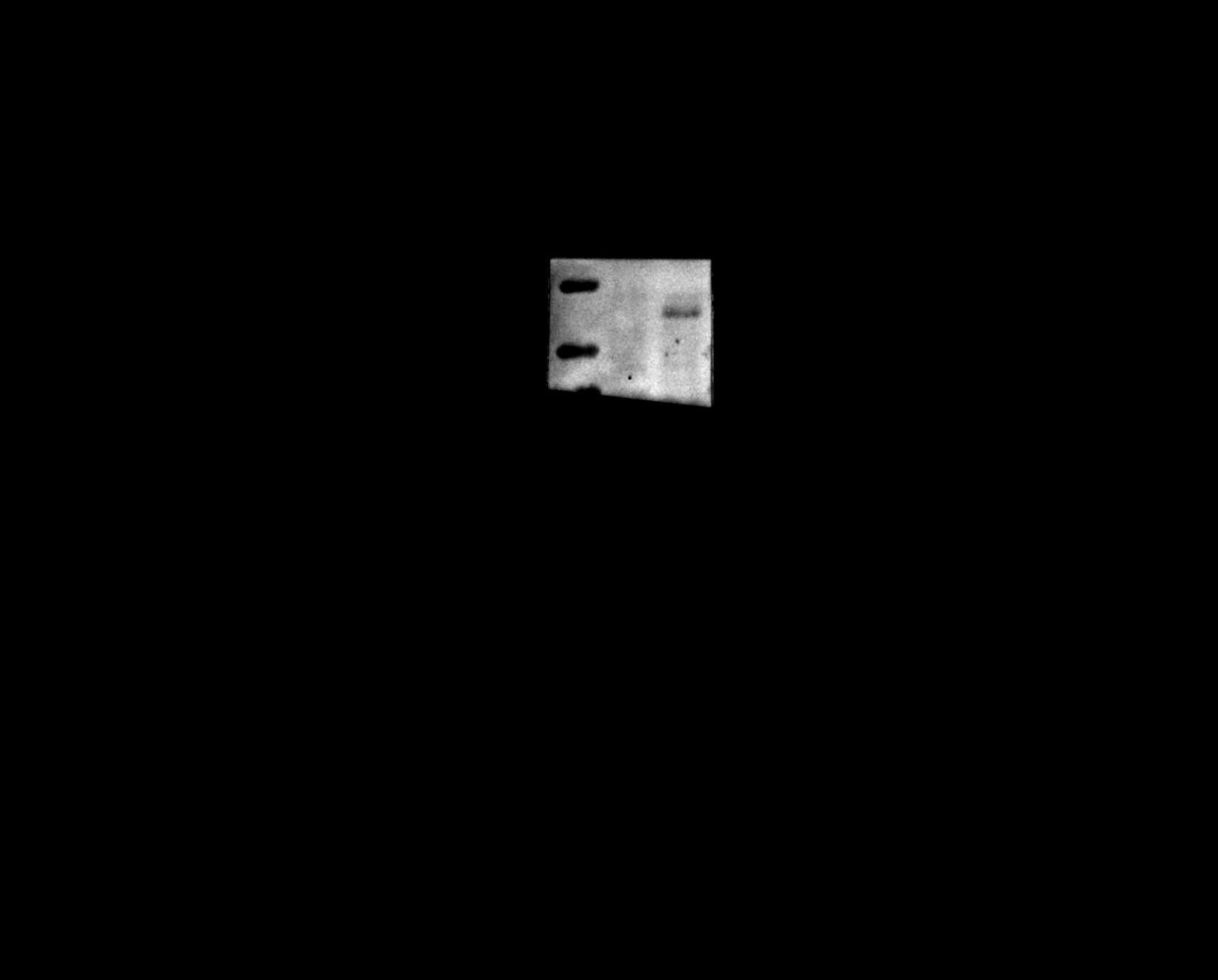

Supplement: Figure 2—source data 2. [file elife-95964-fig2-data2.zip › Figure 2-source data 2/figure 2i/flag-ip.tif]

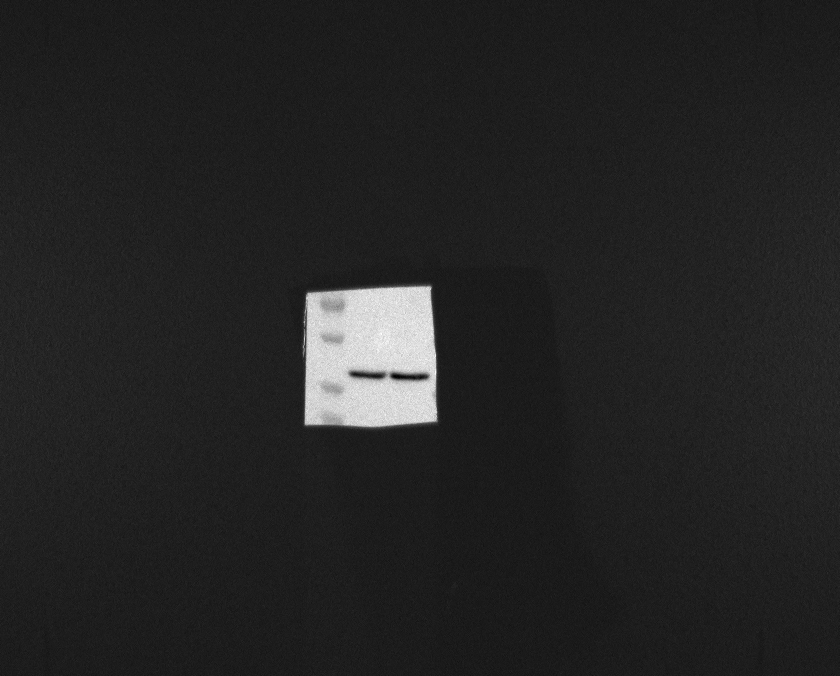

Supplement: Figure 2—source data 2. [file elife-95964-fig2-data2.zip › Figure 2-source data 2/figure 2i/GAPDH.tif]

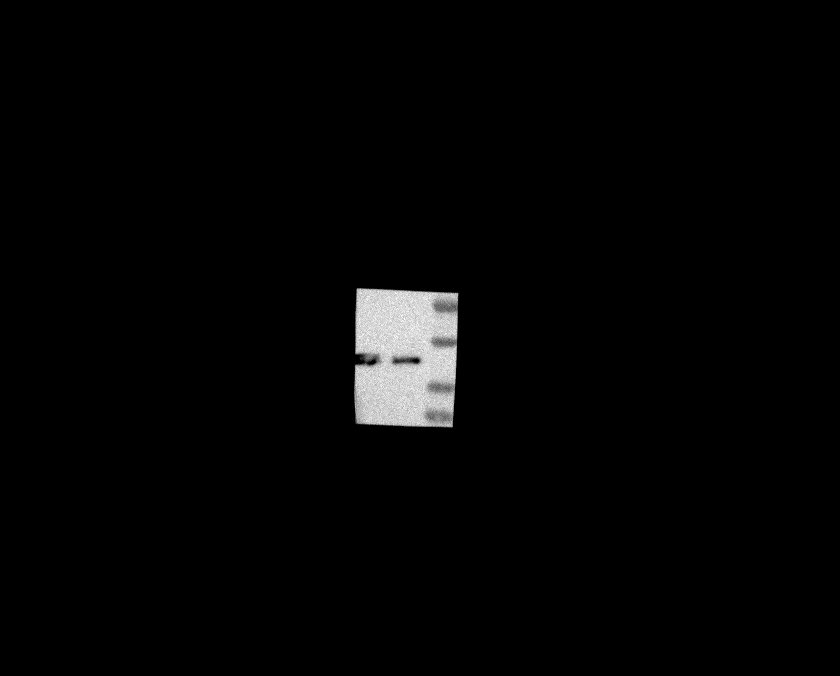

Supplement: Figure 2—source data 2. [file elife-95964-fig2-data2.zip › Figure 2-source data 2/figure 2i/ha-input.tif]

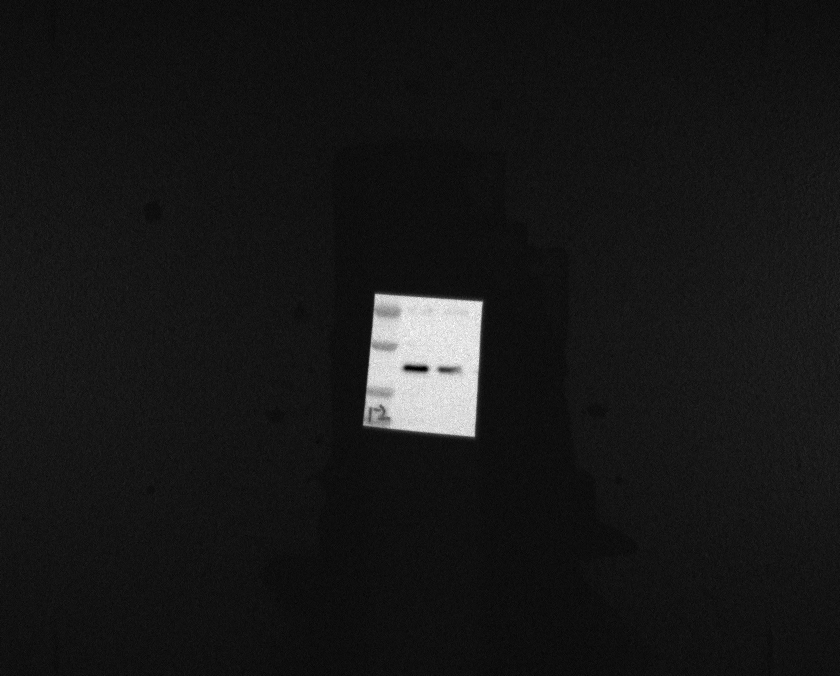

Supplement: Figure 2—source data 2. [file elife-95964-fig2-data2.zip › Figure 2-source data 2/figure 2i/HA-ip.tif]

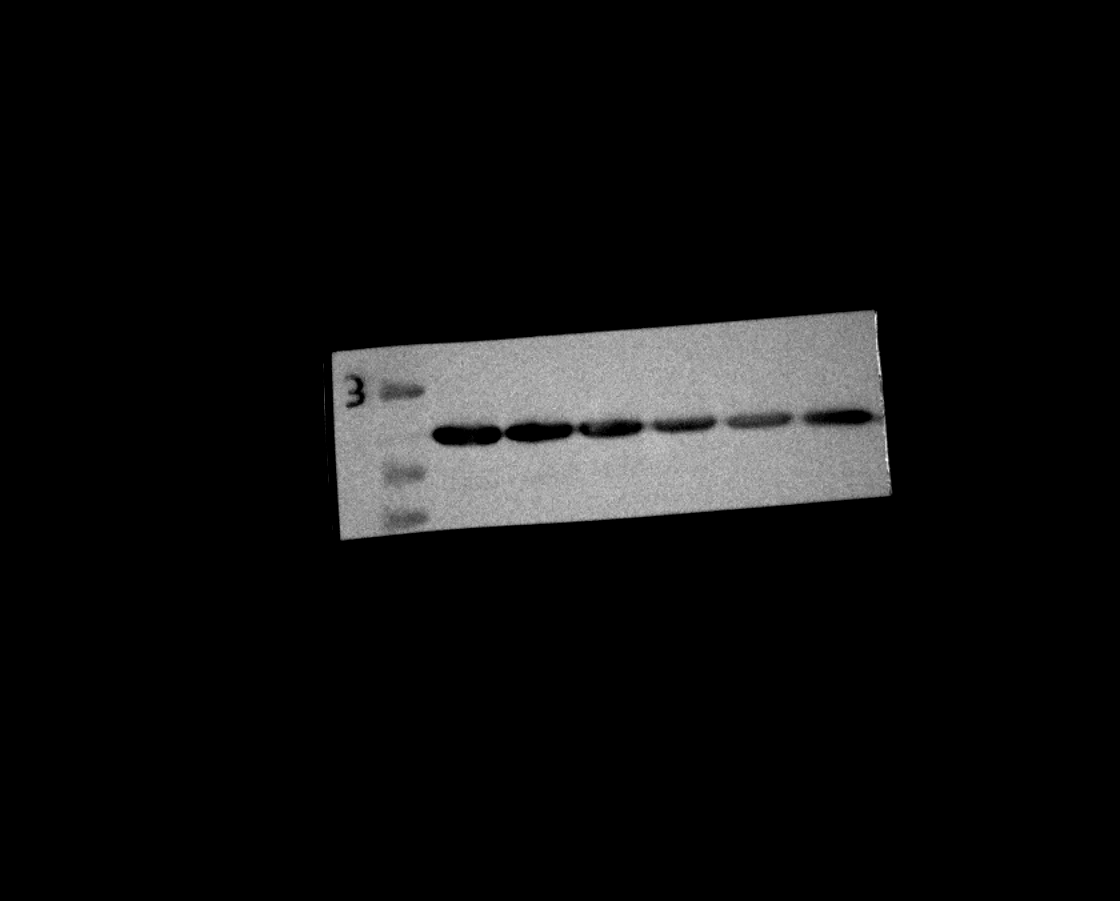

Supplement: Figure 2—source data 2. [file elife-95964-fig2-data2.zip › Figure 2-source data 2/figure 2j/gapdh.tif]

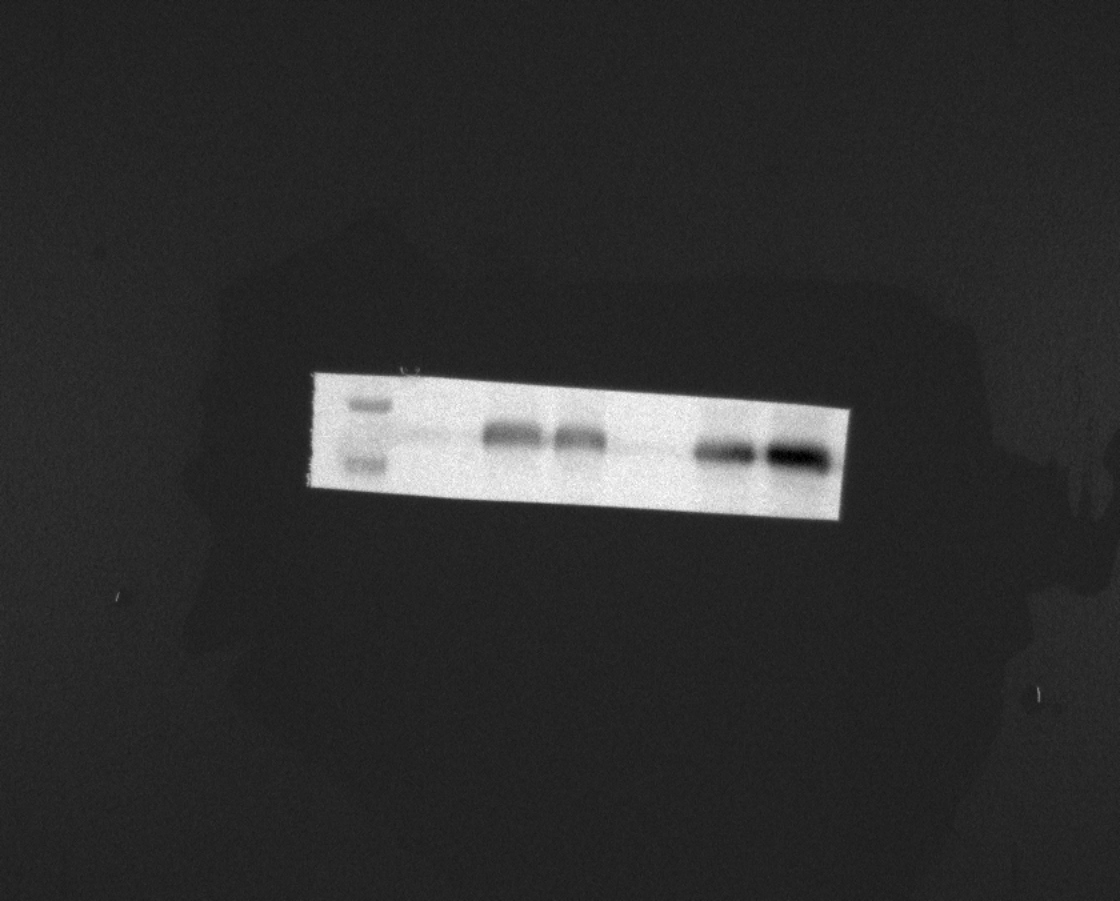

Supplement: Figure 2—source data 2. [file elife-95964-fig2-data2.zip › Figure 2-source data 2/figure 2j/HA.tif]

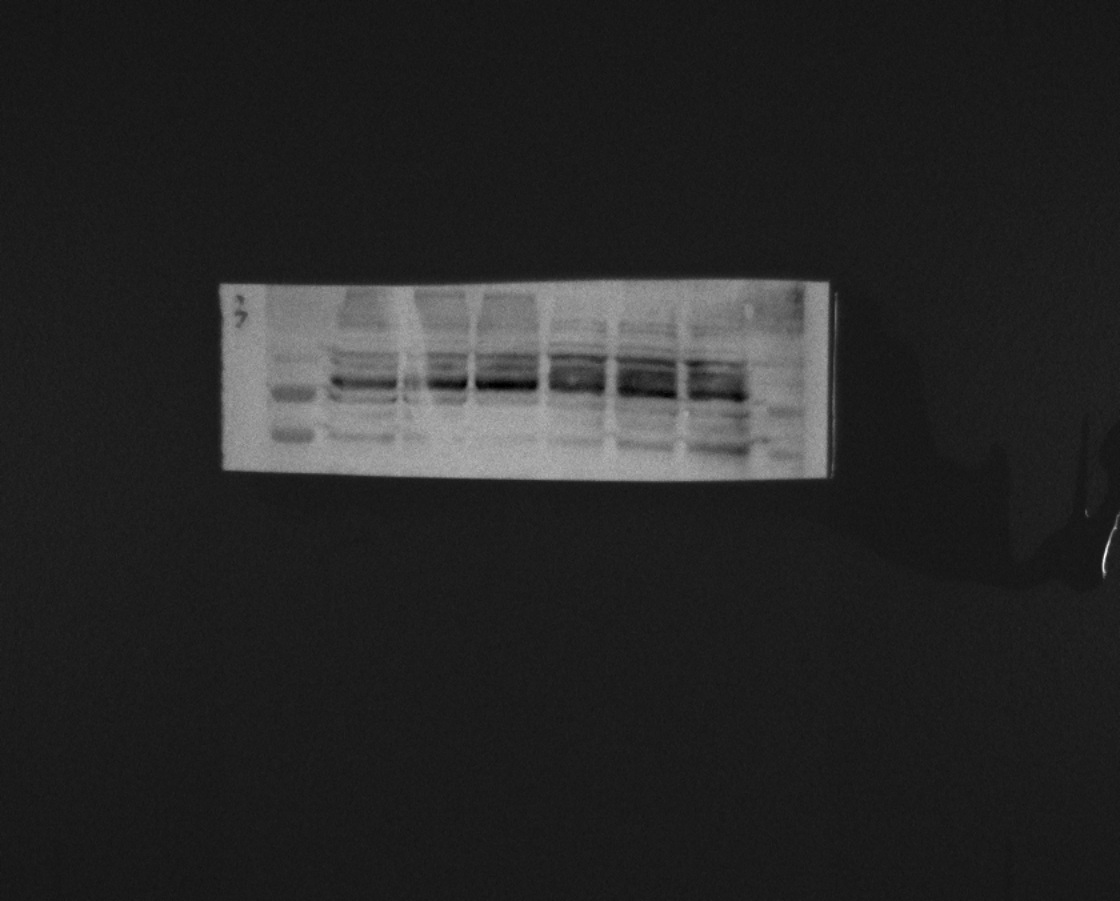

Supplement: Figure 2—source data 2. [file elife-95964-fig2-data2.zip › Figure 2-source data 2/figure 2j/HSF1.tif]

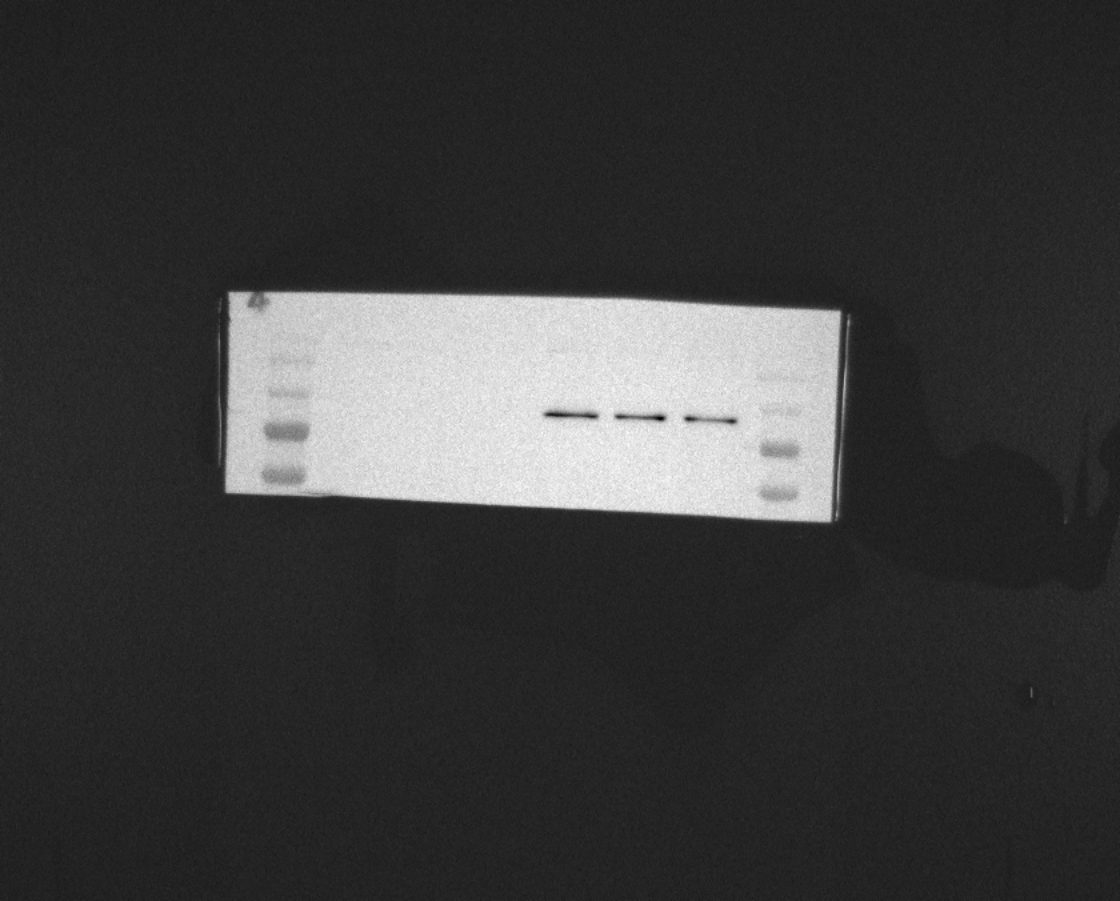

Supplement: Figure 2—source data 2. [file elife-95964-fig2-data2.zip › Figure 2-source data 2/figure 2j/P-HSF1.tif]

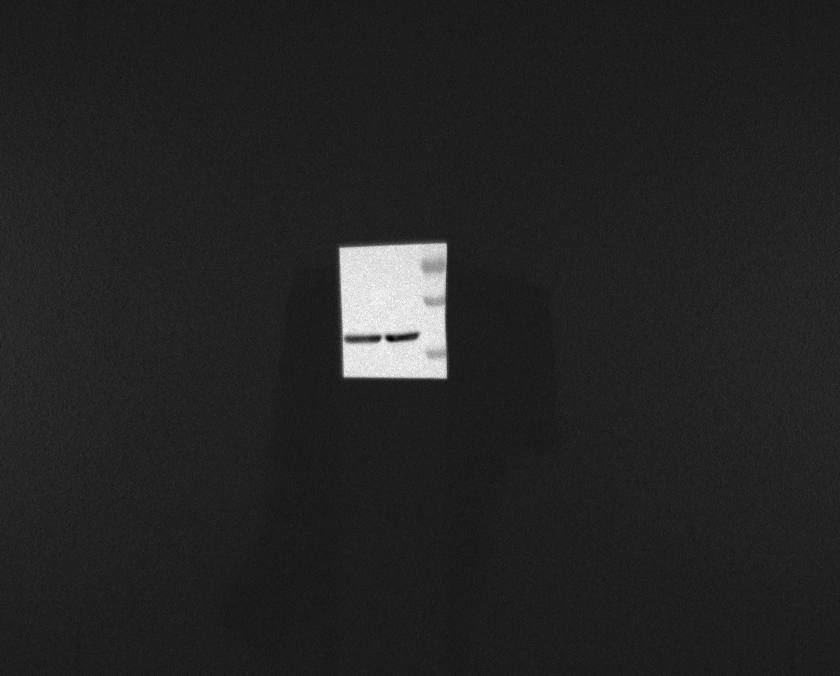

Supplement: Figure 2—source data 2. [file elife-95964-fig2-data2.zip › Figure 2-source data 2/figure 2l/2L-right/gapdh.tif]

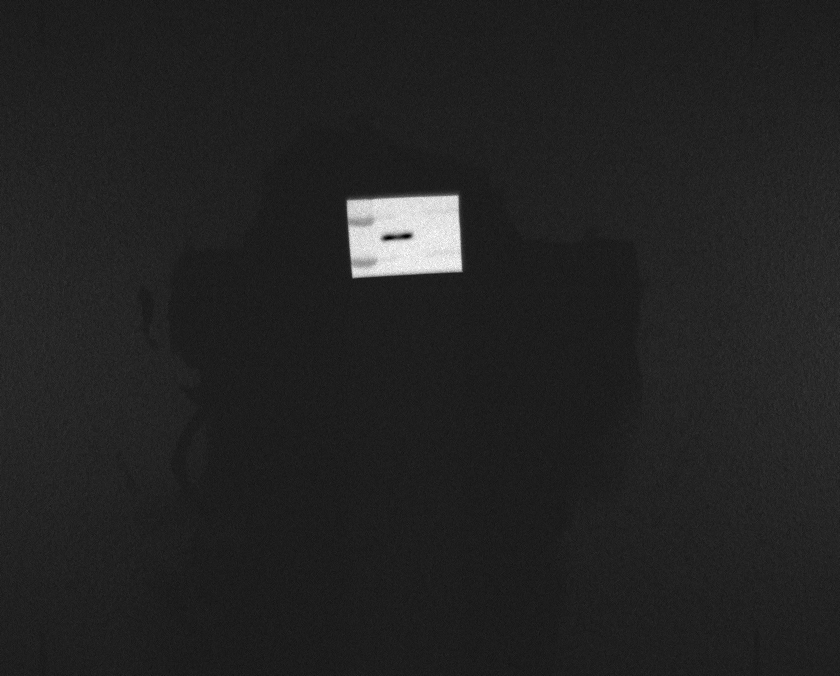

Supplement: Figure 2—source data 2. [file elife-95964-fig2-data2.zip › Figure 2-source data 2/figure 2l/2L-right/HA-input.tif]

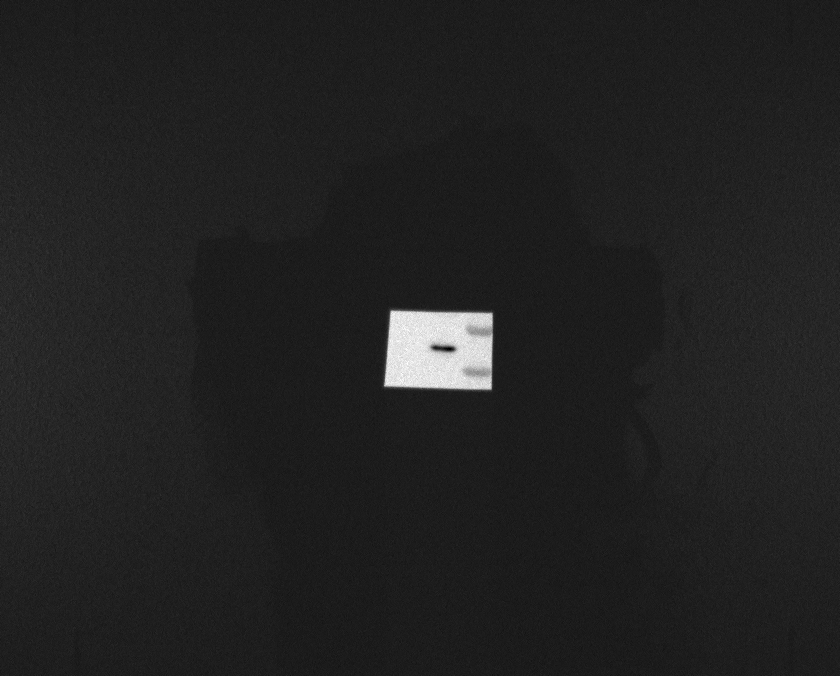

Supplement: Figure 2—source data 2. [file elife-95964-fig2-data2.zip › Figure 2-source data 2/figure 2l/2L-right/HA-IP.tif]

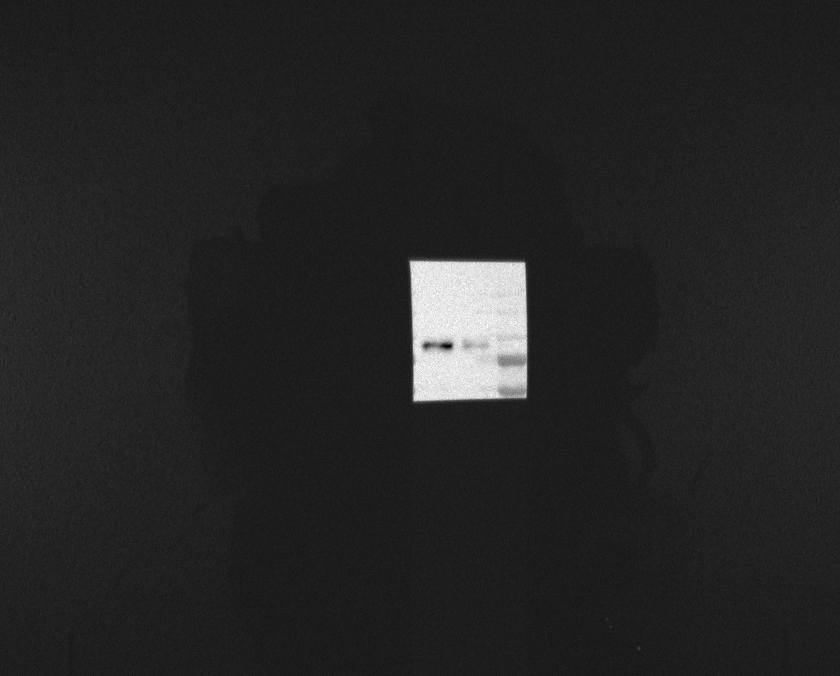

Supplement: Figure 2—source data 2. [file elife-95964-fig2-data2.zip › Figure 2-source data 2/figure 2l/2L-right/Myc-Input.tif]

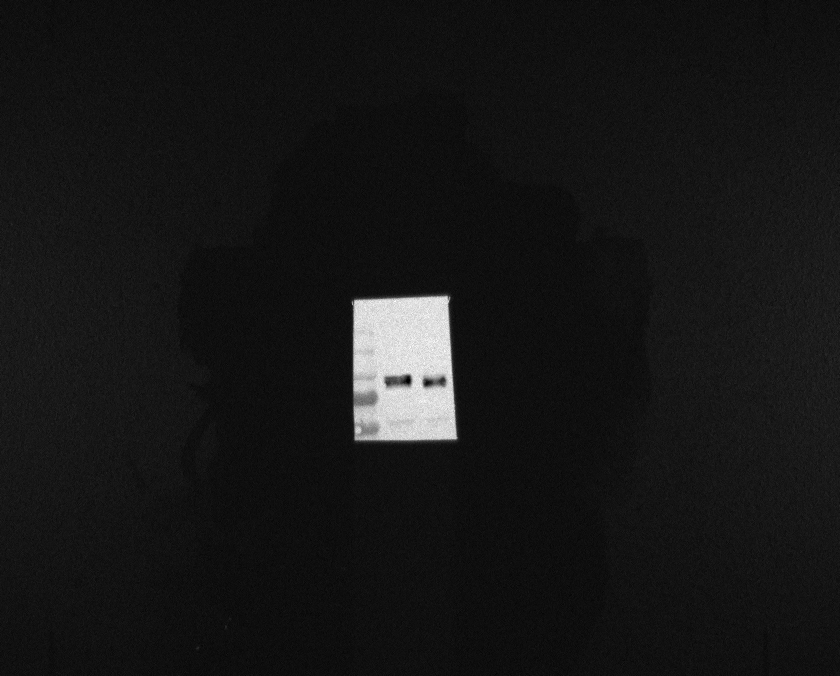

Supplement: Figure 2—source data 2. [file elife-95964-fig2-data2.zip › Figure 2-source data 2/figure 2l/2L-right/Myc-IP.tif]

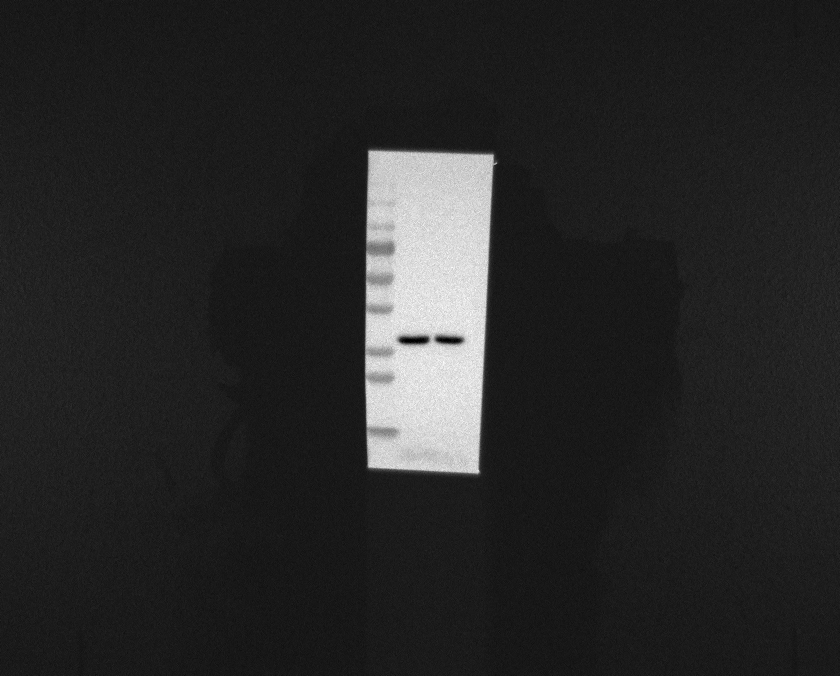

Supplement: Figure 2—source data 2. [file elife-95964-fig2-data2.zip › Figure 2-source data 2/figure 2l/figure 2L-left/GAPDH.tif]

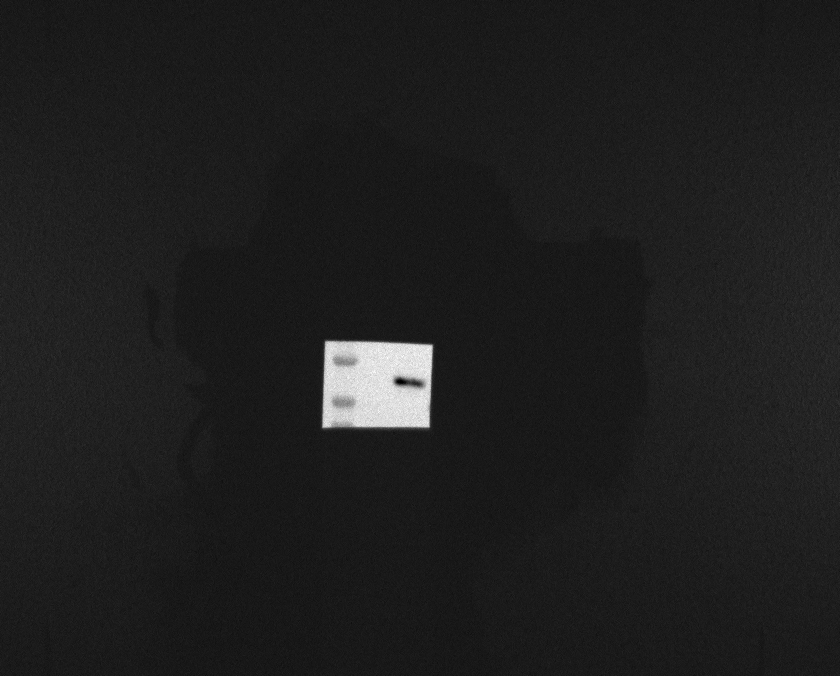

Supplement: Figure 2—source data 2. [file elife-95964-fig2-data2.zip › Figure 2-source data 2/figure 2l/figure 2L-left/HA-input.tif]

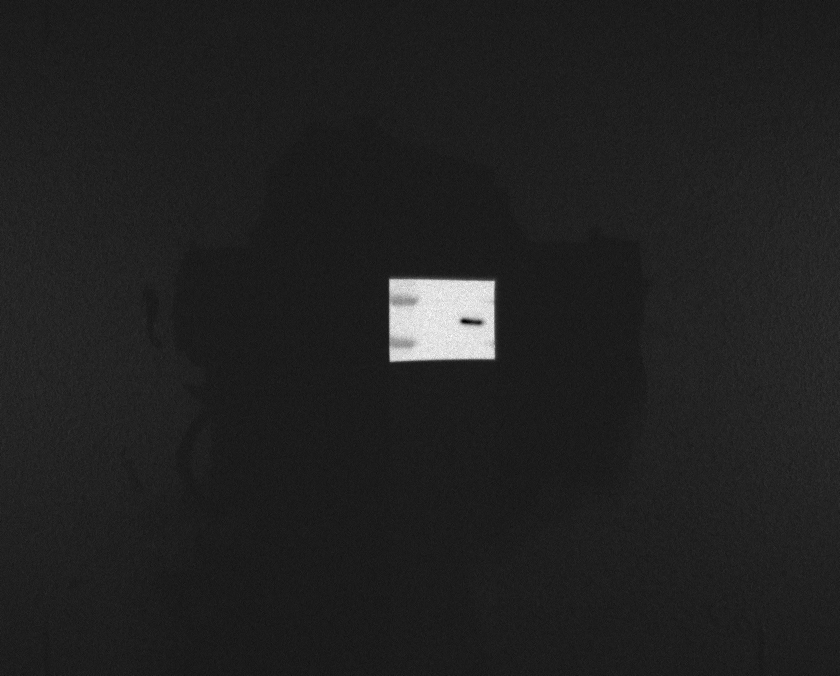

Supplement: Figure 2—source data 2. [file elife-95964-fig2-data2.zip › Figure 2-source data 2/figure 2l/figure 2L-left/HA-IP.tif]

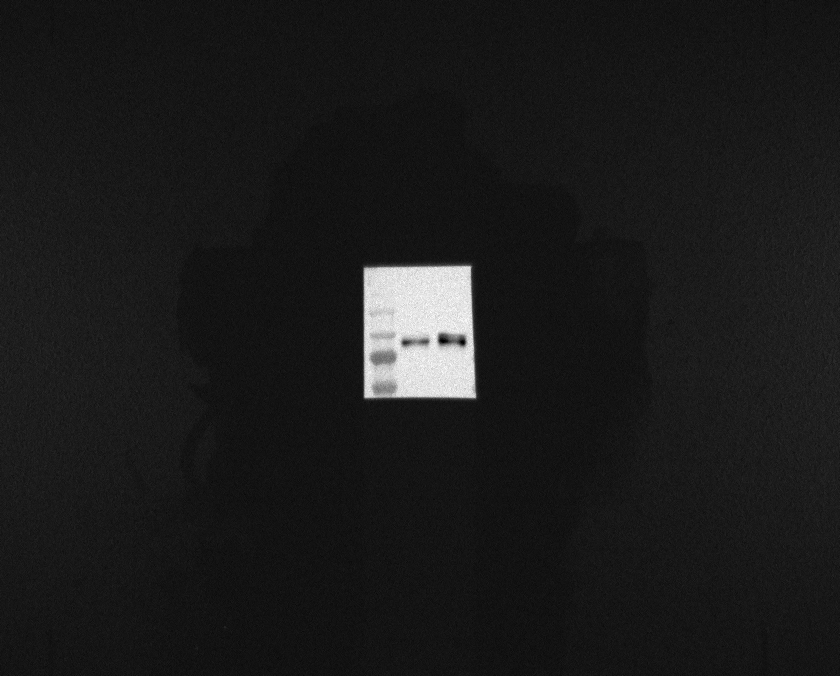

Supplement: Figure 2—source data 2. [file elife-95964-fig2-data2.zip › Figure 2-source data 2/figure 2l/figure 2L-left/HSF1-input.tif]

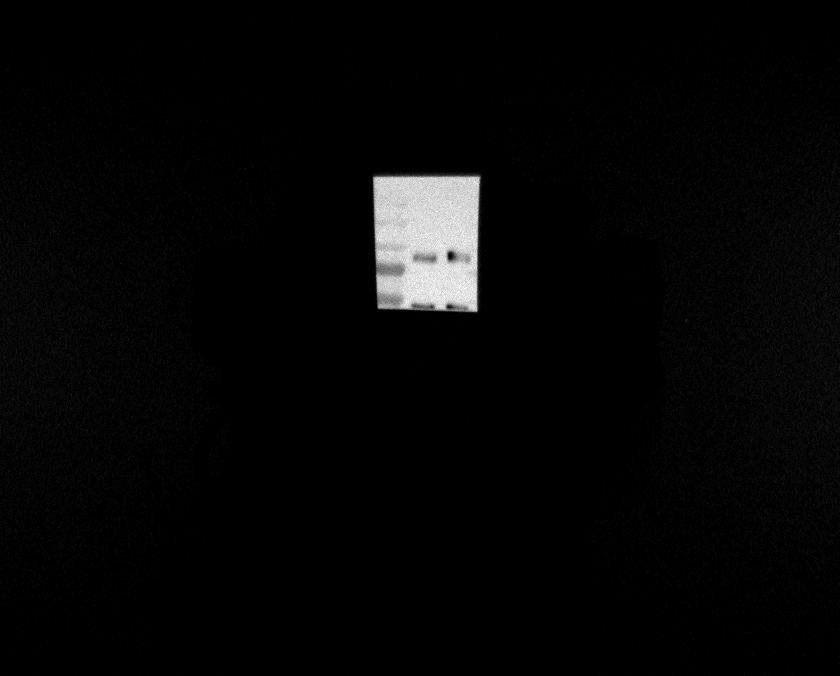

Supplement: Figure 2—source data 2. [file elife-95964-fig2-data2.zip › Figure 2-source data 2/figure 2l/figure 2L-left/HSF1-IP.tif]

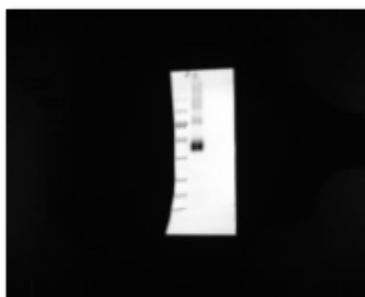

flag-right

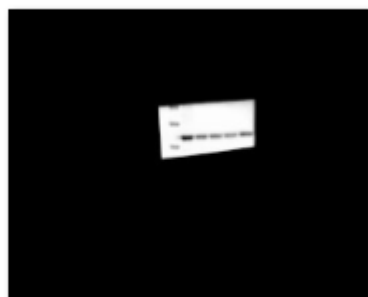

gapdh-left

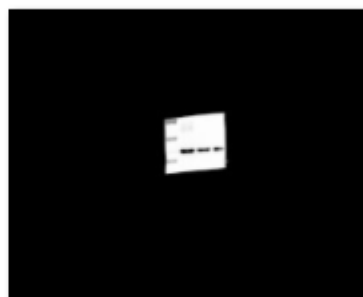

gapdh-right

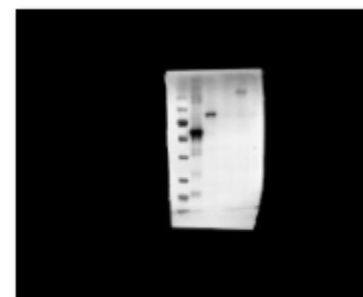

SIV serum-left

Supplement: Figure 3—source data 1. [file elife-95964-fig3-data1.pdf]

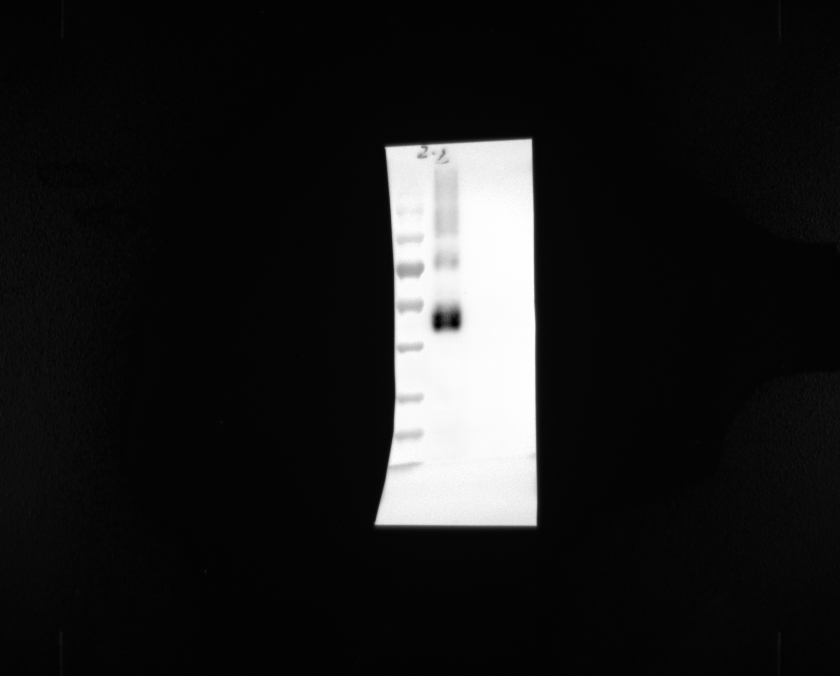

Supplement: Figure 3—source data 2. [file elife-95964-fig3-data2.zip › Figure 3-source data 2/flag-right.tif]

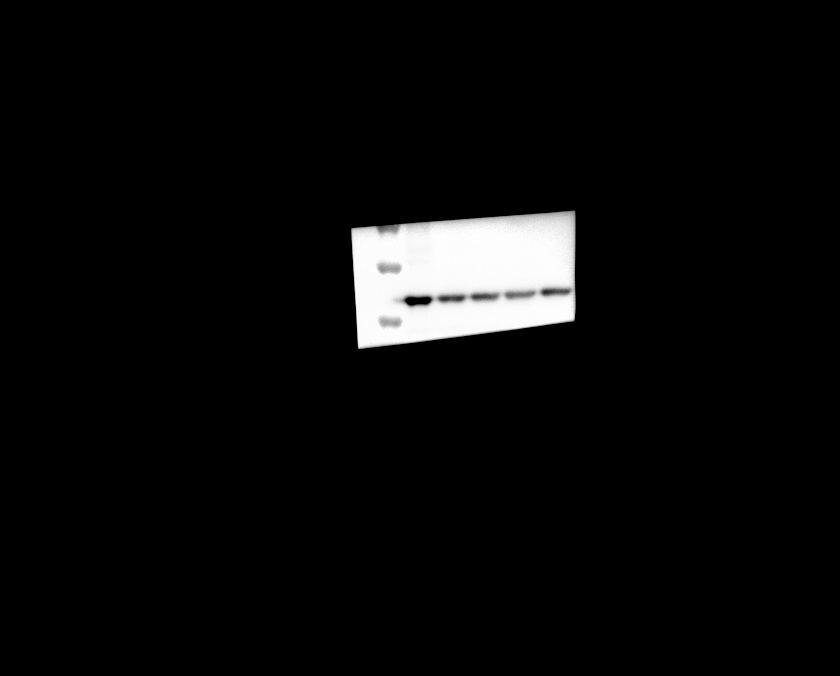

Supplement: Figure 3—source data 2. [file elife-95964-fig3-data2.zip › Figure 3-source data 2/gapdh-left.tif]

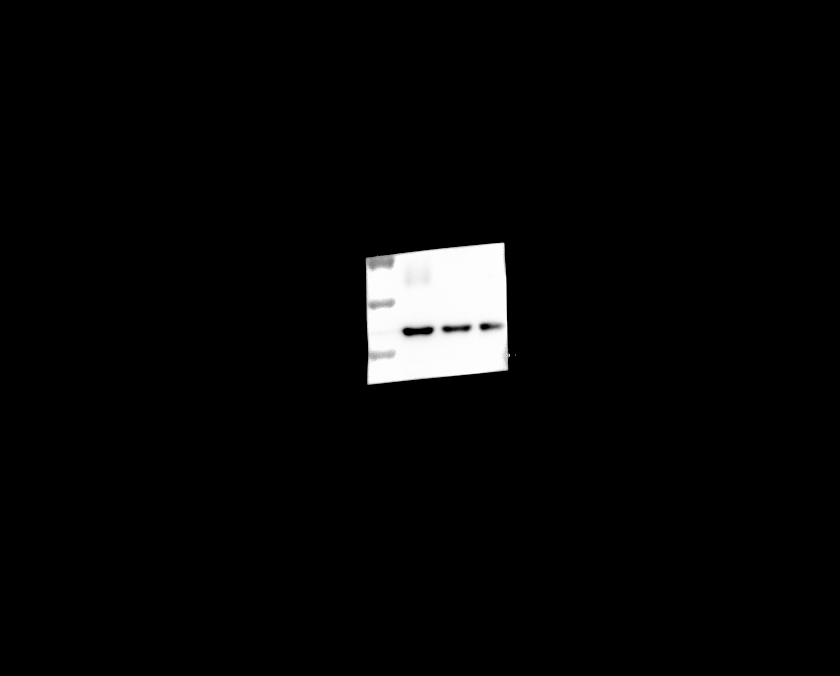

Supplement: Figure 3—source data 2. [file elife-95964-fig3-data2.zip › Figure 3-source data 2/gapdh-right.tif]

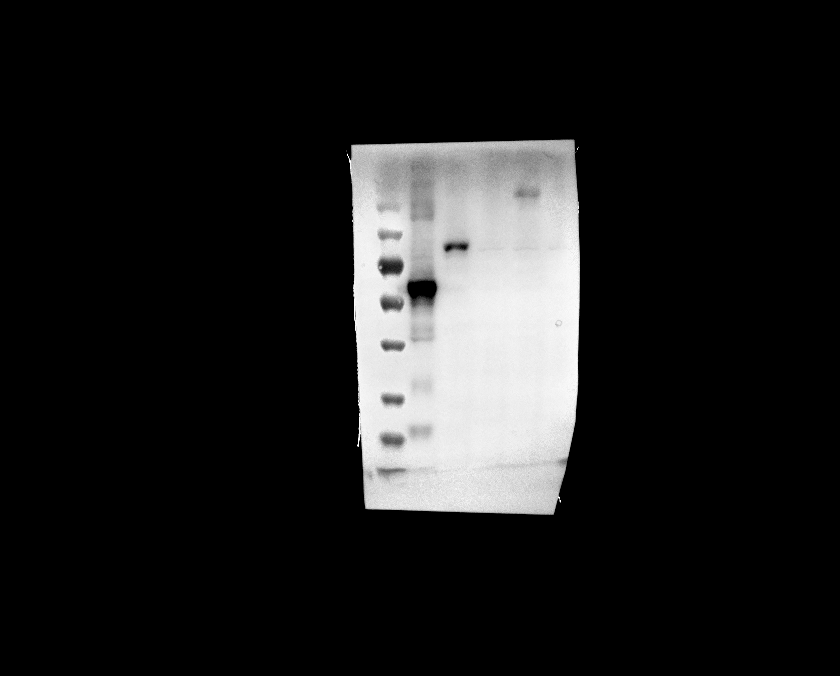

Supplement: Figure 3—source data 2. [file elife-95964-fig3-data2.zip › Figure 3-source data 2/SIV serum-left.tif]
