# Supplementary material for: Purging viral latency by a bifunctional HSV-vectored therapeutic vaccine in chronically SIV-infected macaques
Source: eLife. 2025 Apr 23;13:RP95964. doi: 10.7554/eLife.95964 (PMC12017772; doi:10.7554/eLife.95964)
Supplement: Figure 2—source data 1. [file elife-95964-fig2-data1.pdf]

Figure 2A

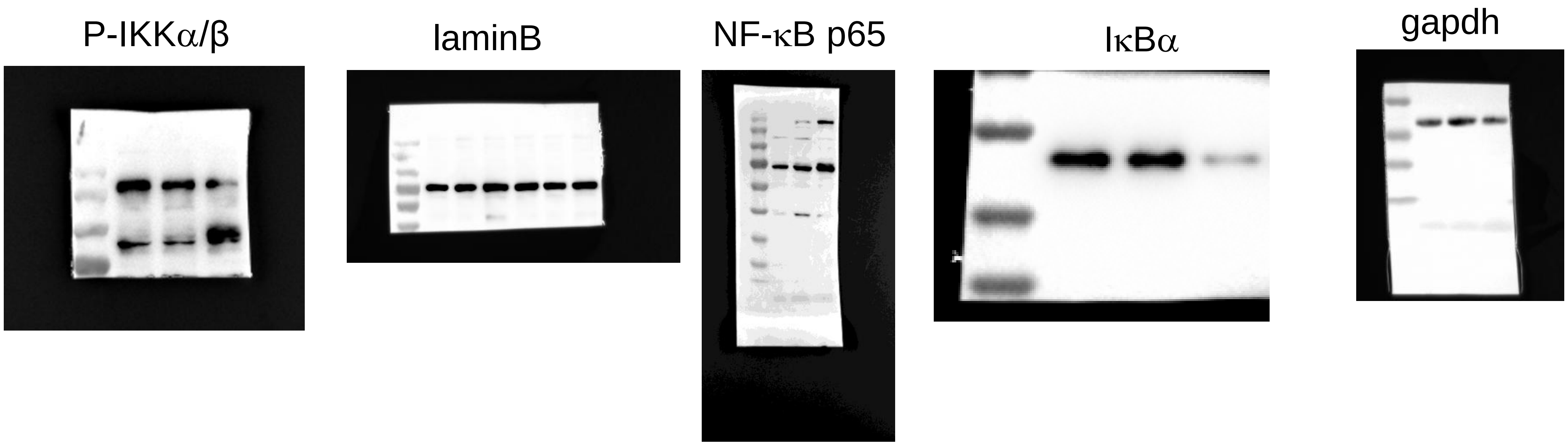

Figure 2B

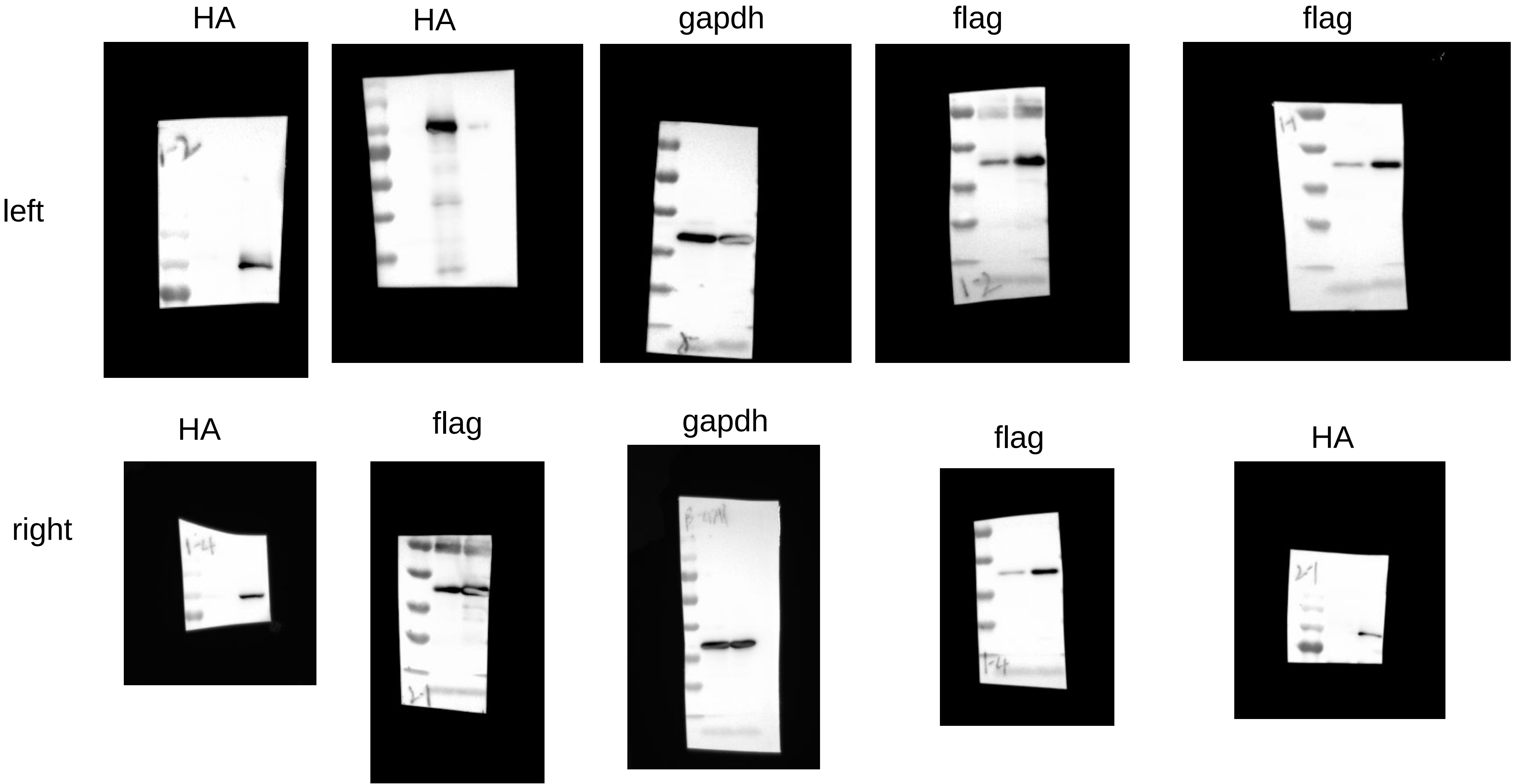

Figure 2G

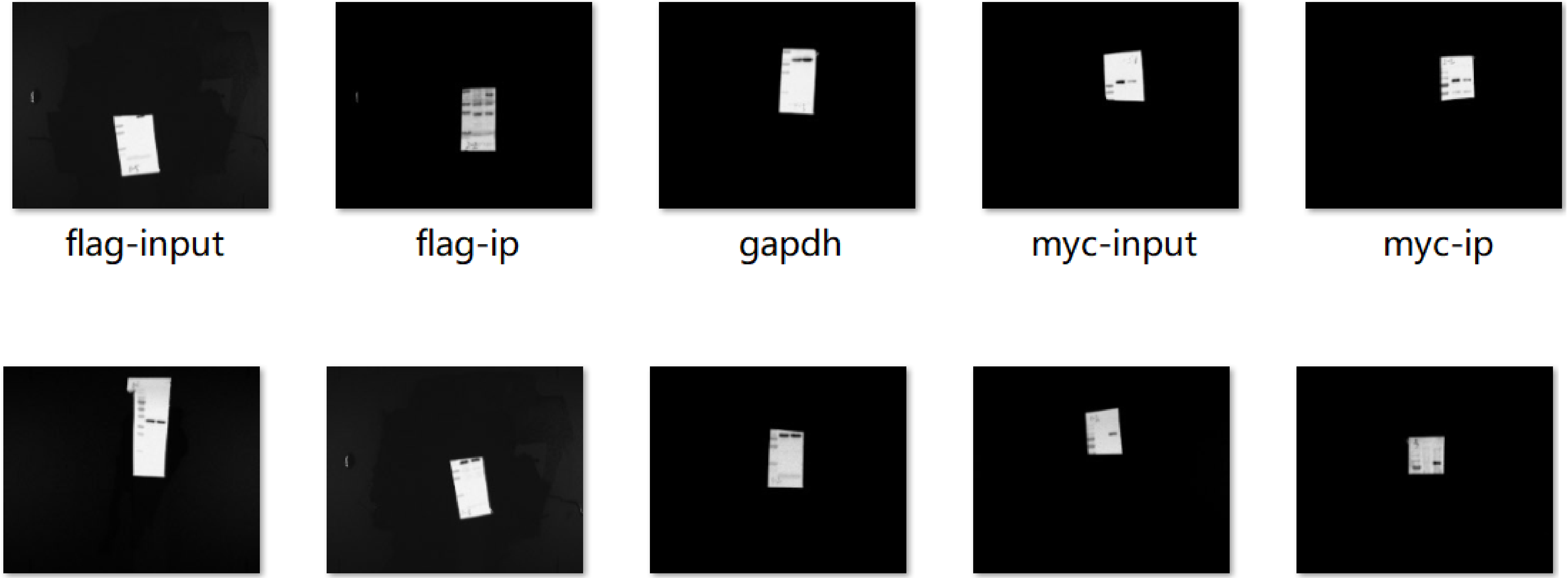

left

flag-input | flag-ip | gapdh | myc-input | myc-ip

right

flag-input | flag-ip | gapdh | myc-input | myc-ip

Figure 2H

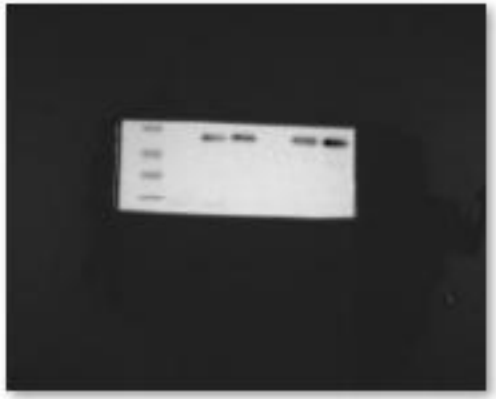

Flag

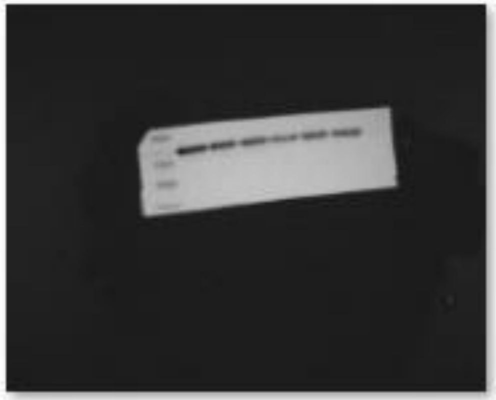

GAPDH

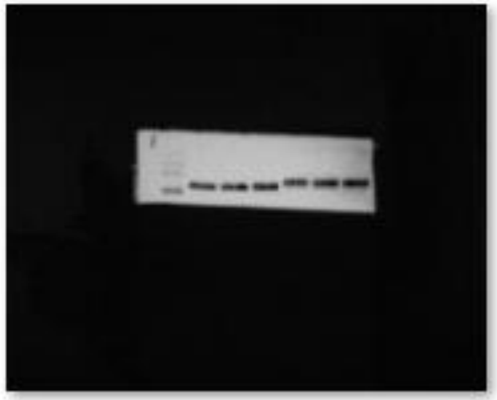

HSF1

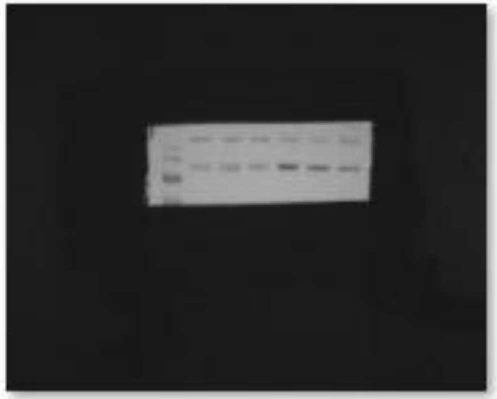

P-HSF1 (Ser 320)

Figure 2I

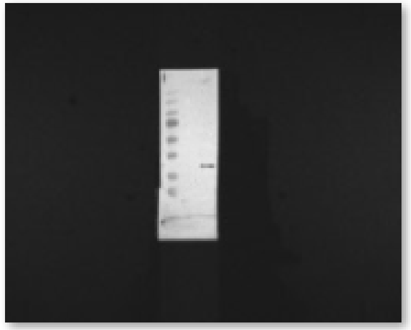

flag-input

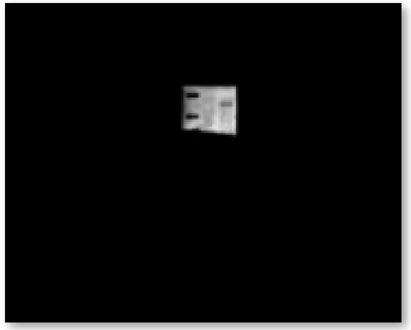

flag-ip

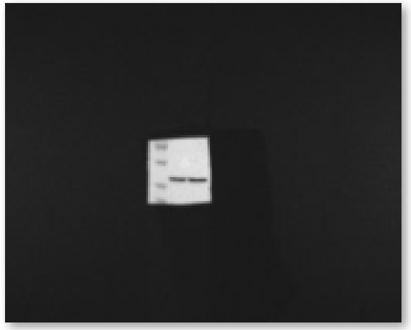

GAPDH

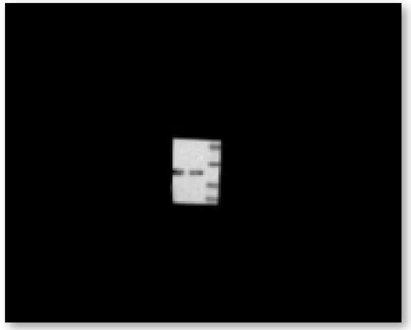

ha-input

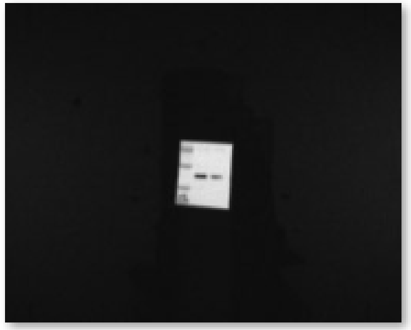

HA-ip

Figure 2J

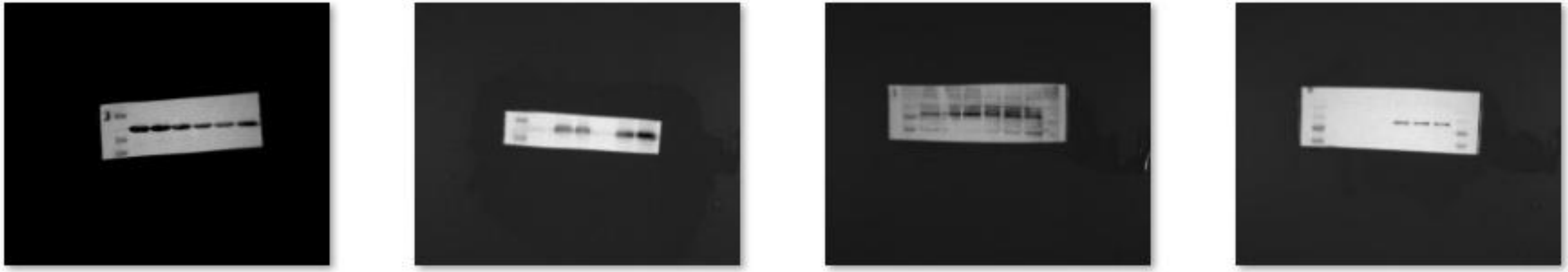

gapdh HA HSF1 P-HSF1

Figure 2K

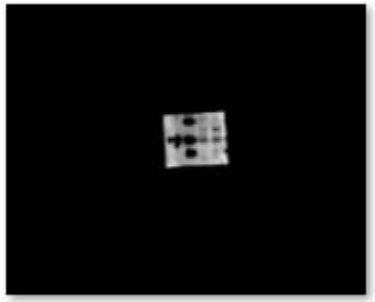

FALG-ip

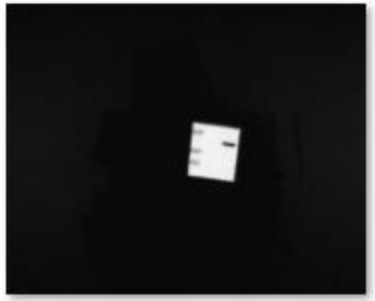

FLAG-input

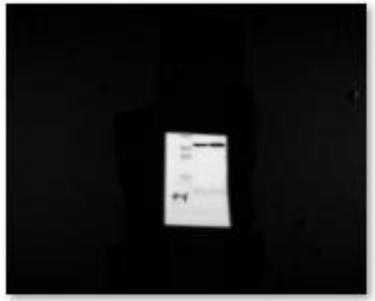

GAPDH

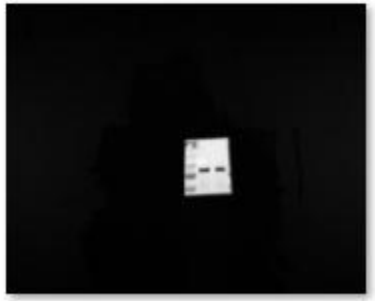

MYC-IP

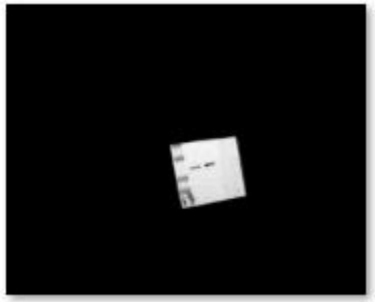

PP1a-input

Figure 2L

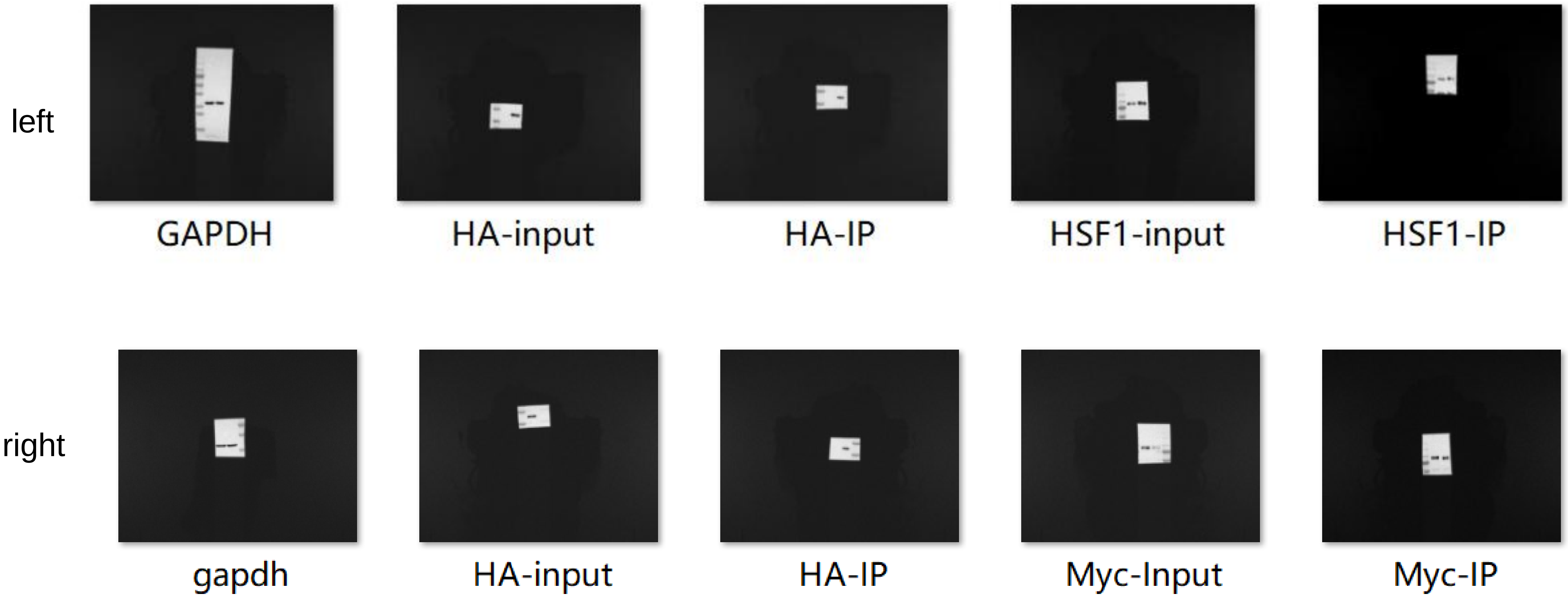
left
GAPDH
HA-input
HA-IP
HSF1-input
HSF1-IP
right
gapdh
HA-input
HA-IP
Myc-Input
Myc-IP
