# Supplementary material for: Purging viral latency by a bifunctional HSV-vectored therapeutic vaccine in chronically SIV-infected macaques
Source: eLife. 2025 Apr 23;13:RP95964. doi: 10.7554/eLife.95964 (PMC12017772; doi:10.7554/eLife.95964)
Supplement: Supplementary file 1. — (a) Immunoprecipitation-mass spectrometry (IP-MS) analysis. (b) Baseline information for the experimental macaques in this study. (c) Antibodies for the intracellular cytokine staining (ICS) assay in this study. (d) The sequences of primers. [file elife-95964-supp1.docx]

**Supplementary files 1**

**Supplementary files 1a. Immunoprecipitation-mass spectrometry (IP-MS) analysis.**

| Accession | -10lgP | Avg. Mass |
| --- | --- | --- |
| P0DMV8\|HS71A_HUMAN | 351 | 70052 |
| P11142\|HSP7C_HUMAN | 337.68 | 70898 |
| P10809\|CH60_HUMAN | 272.28 | 61055 |
| P34931\|HS71L_HUMAN | 261.48 | 70375 |
| P08238\|HS90B_HUMAN | 256.83 | 83264 |
| P34932\|HSP74_HUMAN | 202.19 | 94331 |
| P07900\|HS90A_HUMAN | 198.58 | 84660 |
| O95757\|HS74L_HUMAN | 146.39 | 94512 |
| Q92598\|HS105_HUMAN | 134.36 | 96865 |
| P04792\|HSPB1_HUMAN | 129.61 | 22783 |
| P62136\|PP1A_HUMAN | 186.52 | 37512 |

**Supplementary files 1b. Baseline information for the experimental macaques in this study.**

| **Number** | **Age (y)** | **Gender** | **Viral Load**  **(Log10 copies/mL)** | **CD4 (count/mL)** | **Body Weight（kg）** |
| --- | --- | --- | --- | --- | --- |
| #1 | 11 | M | 5.72 | 83 | 11.25 |
| #2 | 11 | M | 4.05 | 972 | 10.75 |
| #3 | 15 | M | 2.98 | 1819 | 9.10 |
| #4 | 19 | M | 4.35 | 835 | 9.25 |
| #5 | 11 | M | 5.57 | 238 | 14.3 |
| #6 | 11 | M | 3.24 | 599 | 9.60 |
| #7 | 11 | M | 5.99 | 1181 | 6.85 |
| #8 | 18 | F | 4.18 | 767 | 5.65 |
| #9 | 10 | F | 3.82 | 721 | 7.40 |

Note: y: years old; M: male; F: female.

Supplementary files 1c. Antibodies for the ICS assay in this study.

| **Classification** | **Antibodies** |
| --- | --- |
| Mice experiment | Mouse CD3-FITC |
|  | Mouse CD4-BB700 |
|  | Mouse CD8-PECY7 |
|  | Mouse IFN-γ-APC |
|  | Mouse TNF-α-PE |
|  | Mouse IL-2-Violet605 |
|  | Mouse CD44-Ko525 |
|  | Mouse CD62L-BV786 |
|  | Mouse CD107a-BV510 |
|  | Mouse CD49b-PE |
| Monkey experiment | Hu/NHP CD4 FITC L200 |
|  | Hu/NHP CD3 APC SP34-2 |
|  | NHP CD45 PE D058-1283 |
|  | CD8 Percp SK1 |
|  | NHP CD3 BV650 SP34-2 |
|  | Hu/NHP CD4 PE-CF594 L200 |
|  | Hu/NHP CD8 APC-Cy7 RPA-T8 |
|  | Hu/NHP IL-2 APC MQ1-17H12 |
|  | Hu/NHP IFN-γ PE 4S.B3 |
|  | Hu/NHP TNF PE-Cy7 MAb11 |

**Supplementary files 1d. The sequences of primers.**

| **Primers** | **Sequences** |
| --- | --- |
| Tat-F | ATGGAGCCAGTAGATCCTAG |
| Tat-R | TGCTTTGATAGAGAAGCTTG |
| LTR-F | GCCTCAATAAAGCTTGCCTTGA |
| LTR-R | TCCACACTGACTAAAAGGGTCTGA |
| Gag-F | GTCCAGAATGCGAACCCAGA |
| Gag-R | GTTACGTGCTGGCTCATTGC |
| Vpr-F | CCACAAAGGGAGCCATACAATG |
| Vpr-R | TTATGGCTTCCACTCCTGCC |
| Vif-F | CACACAAGTAGACCCTGACCT |
| Vif-R | CCCTACCTTGTTATGTCCTGCT |
| HSF1-F | CCACCTCCACCCCTGAAAAG |
| HSF1-R | GCACCAGCTGCTTCCCTGA |
| β-actin-F | TCACCAACTGGGACGAC |
| β-actin-R | TGTCACGCACGATTTCC |
| SYBR S mac-g238 S | AATACTGTCTGCGTCATCTGG |
| SYBR S mac-g382A | ATGGTGCTGTTGGTCTACTTG |
| tHSV-F | TGCTTGCCTGTCAAACTCTAC |
| tHSV-R | CCTCGGGTGTAACGTTAGAC |
| Ad-F | GAGTTGGCACCCCTATTCGA |
| Ad-R | GTTGCTGTGGTCGTTCTGGT |
| VVtk-F | ATAGATACGGAACGGGACT |
| VV-tk-R | TTGCCATACGCTCACAG |
